# Toxicology and detoxification processing of Fuzi (*Aconitum carmichaelii* Debeaux lateral root): a comprehensive review integrating historical perspectives and modern research

**DOI:** 10.3389/fphar.2026.1750573

**Published:** 2026-04-30

**Authors:** Yi Zhang, Huiwen Bai, Rongyi Zhou, Mumu Wei, Jiaqi Zhang, Xiaolu Yu, Jie Zhang

**Affiliations:** The First Affiliated Hospital of Henan University of Chinese Medicine, Zhengzhou, Henan, China

**Keywords:** *Aconitum carmichaelii* Debeaux, clinical safety, critical assessment, diester–diterpenoid alkaloids, ethnopharmacology, Fuzi, processing (Paozhi), toxicity

## Abstract

Fuzi, the processed lateral root of *Aconitum carmichaelii* Debeaux (Ranunculaceae), is a vital yet highly toxic botanical drug in traditional Chinese medicine. However, its narrow therapeutic window, driven by highly toxic diester-diterpenoid alkaloids (DDAs), necessitates rigorous processing. Adhering to the four pillars of best practice in ethnopharmacology, this review systematically evaluates the toxic components, multi-organ toxicological mechanisms, and detoxification-oriented processing methods of Fuzi, while critically assessing the scientific quality of modern toxicological studies. Current evidence demonstrates that DDAs exert multi-organ toxicity through voltage-gated sodium channel disruption, mitochondrial dysfunction, and oxidative stress, affecting the heart, liver, kidney, nervous system, and reproductive organs. While traditional processing effectively reduces the toxicity by hydrolyzing DDAs into lower-toxicity metabolites, our critical assessment reveals recurrent methodological limitations in contemporary research. These include the inadequate definition of the botanical material, lack of appropriate controls, and reliance on experimental models with questionable clinical relevance. Despite these flaws, current evidence confirms that inadequately processed Fuzi poses severe cardiotoxic and neurotoxic risks, driving ongoing clinical safety challenges. To bridge traditional practices with modern safety requirements, future research must prioritize rigorous experimental designs, toxicological-endpoint-informed quality standards, and prospective clinical pharmacovigilance.

## 1 Introduction

**FIGURE 1:**
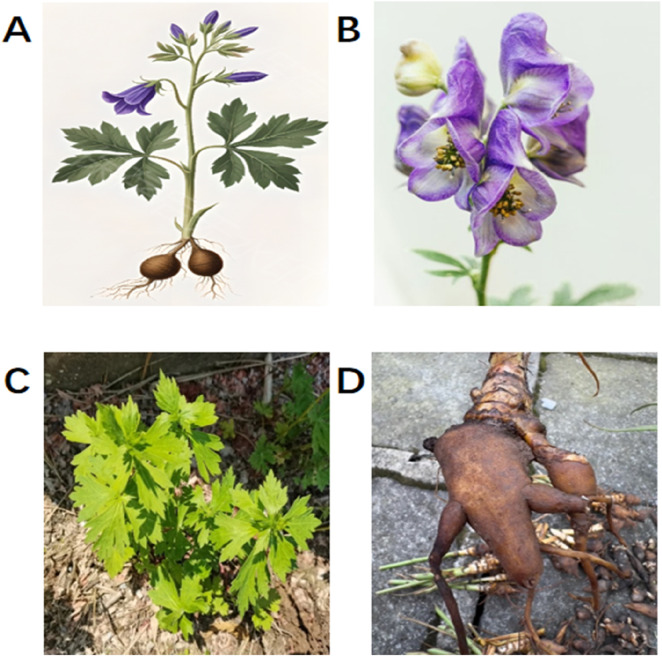
Morphology of *Aconitum carmichaelii* Debeaux. **(A)** Whole plant; **(B)** flowers; **(C)** leaves; **(D)** lateral root (Fuzi).

**FIGURE 2:**
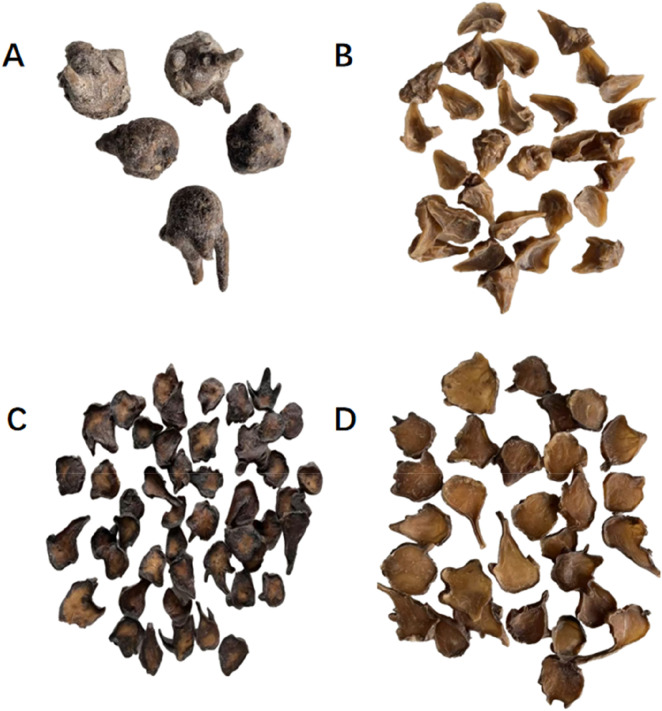
Different forms of Fuzi. **(A)** Salted aconite root (Yan Fuzi); **(B)** raw aconite slices (Sheng Fuzi Pian); **(C)** black sliced aconite (Heishunpian); **(D)** desalted aconite slices (Danfupian).

Fuzi [Aconiti Lateralis Radix Praeparata, the processed lateral root of *Aconitum carmichaelii* Debeaux (Ranunculaceae)], is a botanical drug based in traditional Chinese medicine (TCM) theory for “restoring depleted Yang and rescuing collapse” *(回阳救逆, Huí Yáng Jiù Nì)*. It possesses the effects of tonifying fire to reinforce Yang *(Bu Huo Zhu Yang)* and dispelling cold to alleviate pain *(San Han Zhi Tong)* and is clinically used for conditions such as devastated Yang collapse *(Wang Yang Xu Tuo)*, heart Yang insufficiency *(Xin Yang Bu Zu)*, and pain due to cold–damp bi-syndrome *(Han Shi Bi Tong)* (Figure 1). Modern pharmacological research confirms that its main active metabolites include diterpenoid alkaloids (DAs), flavonoids, and steroidal saponins, which possess anti-inflammatory, analgesic, cardiotonic, and immunomodulatory effects (He Y. X. et al., 2023). Fuzi is widely applied in treating conditions such as chronic heart failure and rheumatic diseases (Bisset, 1981). However, the diester–diterpenoid alkaloids (DDAs) present in Fuzi, such as aconitine (AC), mesaconitine (MA), and hypaconitine (HA), can induce severe toxicity, manifesting as cardiac arrhythmias, nerve paralysis, and multiple organ failure. To balance this “efficacy–toxicity duality,” historical medical practitioners developed over 70 processing (Paozhi) methods. As early as the Han Dynasty, the *Jingui Yuhan Jing* documented detoxification methods such as honey decoction and water boiling. Currently, the Chinese Pharmacopoeia (2020 edition) lists products such as salt-processed Fuzi (Yan Fuzi) and black sliced aconite (Heishunpian, HSP) (Figure 2) as official items, utilizing adjuvants such as salt brine (Danba), licorice (Gancao), and black soybean (Heidou) (Yang, et al., 2021). Despite these established processing methods, clinical poisoning incidents caused by inadequately processed or improperly dosed Fuzi continue to occur, manifesting as lethal cardiac arrhythmias, neurotoxicity, and multi-organ failure (Chung et al., 2021; Shang et al., 2025). However, existing reviews either exclude alkaloid toxicology (Fu et al., 2022), lack formal quality assessment frameworks (He G. et al., 2023), or cover only preclinical cardiotoxicity (Jiang et al., 2022), leaving a comprehensive multi-organ toxicological overview as an unmet need.

In this review, we systematically integrate information from classic Materia Medica, modern research, and pharmacopoeial standards. Our aim is to (1) characterize the principal toxic metabolites and their structure–toxicity relationships; (2) evaluate multi-organ toxicological evidence covering cardiotoxicity, hepatotoxicity, nephrotoxicity, neurotoxicity, developmental toxicity, reproductive toxicity, gastrointestinal toxicity, and subacute/chronic toxicity; (3) analyze the chemical mechanisms underlying toxicity reduction during processing (such as hydrolysis, transesterification, and ion-pair formation); and (4) evaluate the efficiency of both traditional and innovative techniques (such as high-pressure steaming and microbial fermentation) regarding the transformation of DDAs and the retention of bioactive monoester-diterpenoid alkaloids (MDAs) and aminoalcohol-diterpenoid alkaloids (ADAs). The findings provide theoretical support for the safe clinical application of Fuzi and the modernization of its processing technology. Taken together, the persistent occurrence of AC poisoning despite the pharmacopoeial standards underlines the urgent need for toxicity-centered, processing-oriented research on Fuzi.

## 2 Materials and methods

### 2.1 Definition of the botanical drug

The botanical drug investigated in this review is the lateral root of *Aconitum carmichaelii* Debeaux (Ranunculaceae), which is known as Fuzi (附子) in TCM and officially monographed as Aconiti Lateralis Radix Praeparata in the Chinese Pharmacopoeia (ChP, 2020 edition). The taxonomic identity was validated against Kew’s Plants of the World Online (POWO) and the Medicinal Plant Names Service (MPNS). Only preparations unequivocally derived from *A. carmichaelii* Debeaux lateral roots were included; studies on other *Aconitum* species [e.g., *A. kusnezoffii* Rchb. and *A. coreanum* (H.Lév.) Rapaics] were excluded. All auxiliary botanical drugs mentioned as processing adjuvants—*Glycyrrhiza uralensis* Fisch. ex DC. (Fabaceae), *Zingiber officinale* Roscoe (Zingiberaceae), and *Glycine max* (L.) Merr. (Fabaceae)—were likewise validated against POWO/MPNS, and their full taxonomic names are provided at the first mention.

Throughout this manuscript, “botanical drug” replaces “herb” or “herbal medicine;” “metabolites” refers to plant-derived small molecules (such as AC, MA, and HA); and alkaloid subclasses are designated as DDAs, MDAs, and ADAs, following the current phytochemical convention. Processed Fuzi products [such as HSP, Baifupian (BFP), and Danfupian) are described by their pharmacopoeial names, with the processing type specified.

### 2.2 Literature search strategy

This review draws on two complementary source categories: (i) modern experimental and clinical literature on Fuzi toxicology and processing science and (ii) classical Chinese Materia Medica texts documenting the historical evolution of the processing methods.

Modern literature: The PubMed/MEDLINE, Web of Science, Embase, CNKI, Wanfang Data, and VIP Chinese Journals Database were searched from inception to 31 December 2025. English-language searches combined the following terms:

- (“*Aconitum carmichaelii*” OR “Aconiti Lateralis Radix Praeparata” OR “Fuzi” OR “processed aconite”)
- AND (“toxicity” OR “toxicology” OR “poisoning” OR “cardiotoxicity” OR “hepatotoxicity” OR “nephrotoxicity” OR “neurotoxicity” OR “developmental toxicity” OR “reproductive toxicity” OR “genotoxicity” OR “adverse effects”)
- AND/OR (“processing” OR “Paozhi” OR “detoxification” OR “hydrolysis” OR “steaming” OR “boiling” OR “fermentation” OR “alkaloid transformation”).

For Chinese-language databases, the following equivalent terms were used: (“附子” OR “制附子” OR “黑顺片” OR “白附片” OR “淡附片”) AND (“毒性” OR “毒理” OR “中毒” OR “安全性” OR “炮制” OR “减毒” OR “水解” OR “生物碱转化”). Reference lists of the retrieved articles and the “cited by” function of PubMed and Web of Science were used to trace additional relevant studies.

Historical sources: Classical Materia Medica texts and formularies from the Han Dynasty (202 BCE) to the Republic of China era (1912–1949) were surveyed using a targeted bibliographic approach because these primary sources are not indexed in electronic databases. Texts were accessed through critical scholarly editions and the Chinese Medical Classics Database (中医古籍数据库). Selection was guided by established indices of Chinese Paozhi literature. Only texts with verifiable authorship, uncontested dynasty attribution, and at least one identifiable processing method with sufficient procedural detail were included. The processing terminology was translated following the WHO International Standard Terminologies on Traditional Medicine in the Western Pacific Region (2007); where no direct English equivalent existed, transliterated *Pinyin* was retained alongside a descriptive gloss.

In addition to the original experimental and clinical studies, a targeted search for review articles on *Aconitum carmichaelii* and Fuzi published between January 2022 and December 2025 was conducted using the same databases. The purpose of this supplementary search was to identify state-of-the-art reviews addressing Fuzi toxicology, processing, and clinical safety; in order to contextualize the scope and novelty of the present work, see Section 5.3 (comparison with existing reviews).

### 2.3 Inclusion and exclusion criteria

Studies were included if they fulfilled the following criteria (modern studies):

1. Investigated Fuzi (*A. carmichaelii* Debeaux lateral root) or its defined metabolites as the test item.
2. Reported original data on at least one of the following: acute or chronic toxicity, target-organ toxicity, developmental/reproductive toxicity, genotoxicity, clinical poisoning, or quantitative changes in the DDA/MDA/ADA content before and after processing.
3. Provided sufficient methodological detail (the model, dose/concentration range, route, duration, and controls) to permit quality assessment.
4. Published as a full peer-reviewed article in English or Chinese.

Studies were excluded based on the following criteria:

1. Other Aconitum species without a clear separation from *A. carmichaelii* Debeaux.
2. Complex polyherbal prescriptions where toxic effects could not be attributed to Fuzi or its metabolites.
3. Reviews, editorials, conference abstracts, or case reports lacking dose/preparation details.
4. Studies reporting only pharmacological benefits without toxicological or detoxification endpoints.
5. Processing studies without a clearly defined method or lacking any chemical or biological measure of detoxification.

Historical sources were included if they contained at least one explicit Fuzi processing description that was classifiable into one of the four standard categories (preparation/*Xiuzhi Fa*, water-based/*Shui Zhi Fa*, fire-based/*Huo Jiagong*, and fire–water co-processing/*Huo Shui Jiagong*) and excluded if the authorship/dating was considered spurious or the text merely listed Fuzi as part of a prescription without processing instructions.

### 2.4 Data extraction, quality assessment, and critical appraisal

Titles and abstracts were screened independently by two reviewers; full texts of potentially eligible articles were assessed for inclusion, with disagreements resolved by a third reviewer. Data were extracted into structured spreadsheets covering the following factors: the botanical drug identity and processing method; extract type and chemical characterization (e.g., quantified DDA/MDA content and metabolite purity); experimental model; dose range, route, duration, and controls; primary toxic endpoints and quantitative results; and proposed mechanisms. For processing studies, additional fields recorded the processing conditions (temperature, duration, and adjuvant type/ratio) and the analytical method used for alkaloid quantification (such as HPLC and UPLC-MS/MS). For historical sources, the dynasty, source title, processing category, high-level method summary, and any quality-control endpoints that were mentioned were recorded; these data are listed in Table 3, with the full original quotations provided in Supplementary Table S1.

The scientific quality of each included modern study was critically assessed in line with the four pillars of best practice in ethnopharmacology and the ConPhyMP guidelines focusing on the following: (a) the definition and characterization of the test material; (b) appropriateness of the experimental design and controls; (c) completeness of pharmacological/toxicological parameter reporting; and (d) reproducibility of the processing methods and analytical rigor. For historical sources, the bibliographic reliability and specificity of the processing description were evaluated. Deficiencies were noted qualitatively in the relevant text sections rather than via numerical scoring, and cross-cutting limitations are discussed in a dedicated subsection (Section 5: *Limitations and future research needs*).

## 3 Review of Fuzi toxicity studies

### 3.1 Toxic metabolites of Fuzi

The toxicological significance of Fuzi (*Aconitum carmichaelii* Debeaux) is primarily attributable to its C19-DDAs, including AC, MA, and HA as the principal toxic metabolites responsible for the severe cardiotoxic and neurotoxic effects associated with Fuzi ingestion (He G. et al., 2023; Liang et al., 2025; Fu et al., 2022). Since systematic phytochemical and toxicological investigations of *Aconitum* species commenced in the mid-twentieth century, convergent evidence has consistently demonstrated that the DDA subclass constitutes the critical toxic fraction, with traditional processing methods involving hydrolysis of the C-8 acetyl ester bond of DDAs to yield the monoester-diterpenoid alkaloid (MDA) counterparts—benzoylaconine (BAC), benzoylmesaconine (BMA), and benzoylhypaconine (BHA)—a transformation that reduces the toxicity by approximately three orders of magnitude (Yang et al., 2018; Liang et al., 2025). Further hydrolysis of the C-14 benzoyl group generates the essentially nontoxic ADAs, such as aconine, mesaconine, and hypaconine, which accumulate with prolonged processing (Liang et al., 2025).

**FIGURE 3:**
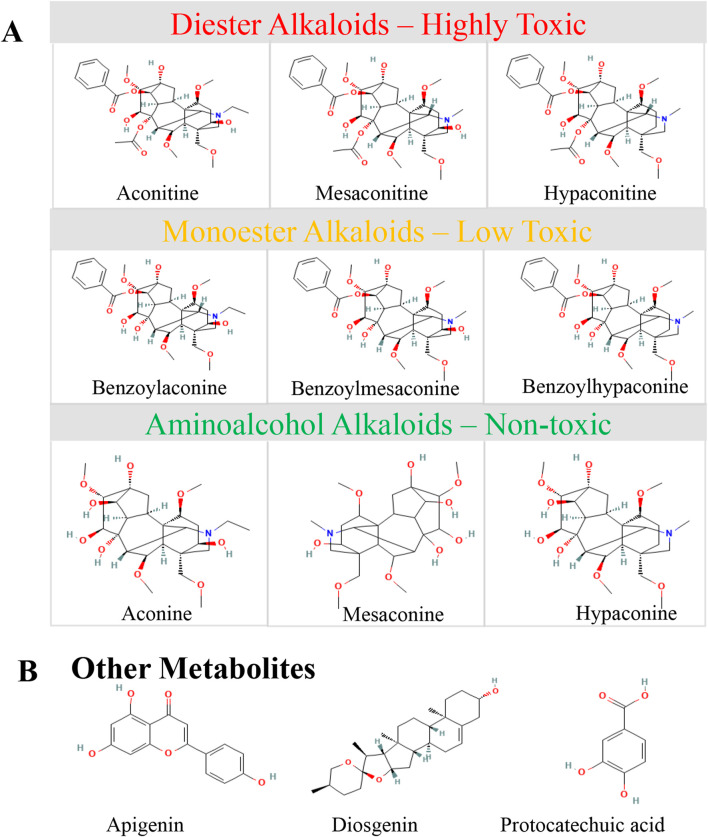
Representative chemical structures and the structure–toxicity relationship of Fuzi metabolites. **(A)** DDAs (highly toxic): aconitine, mesaconitine, hypaconitine; MDAs (low-toxicity): benzoylaconine, benzoylmesaconine, benzoylhypaconine; ADAs (non-toxic): aconine, mesaconine, hypaconine. **(B)** Other bioactive metabolites: apigenin, diosgenin, and protocatechuic acid.

In total, three distinct structural classes of DAs with fundamentally different toxicity profiles have now been identified in Fuzi and its processed products: (1) DDAs (highly toxic: LD_50_ ∼1 mg/kg–6 mg/kg i.g. in mice), which are the dominant toxic metabolites of raw Fuzi; (2) MDAs (moderately low toxicity: LD_50_ ∼0.8 mg/kg–1.5 g/kg i.g. in mice), which are the principal alkaloid metabolites of correctly processed Fuzi products such as HSP and Paofupian; (3) ADAs (essentially non-toxic), which accumulate with prolonged processing (Yang et al., 2018; Liang et al., 2025). This structure–toxicity relationship provides the pharmacological rationale for the traditional Chinese pharmacopoeial requirement that Fuzi must undergo standardized processing before clinical use (see Table 2; Figure 3). Accordingly, our subsequent discussion focuses on DDA-driven toxicity and their detoxification-relevant transformation products.

### 3.2 Historical records of Fuzi toxicity

As a typical TCM botanical drug exhibiting a “dual edge of efficacy and toxicity,” the understanding of toxicity in Fuzi has been systematically recorded within Materia Medica literature since the Han Dynasty. The earliest record appears in *Shennong’s Classic of Materia Medica* (*Shennong Bencao Jing*), which classified botanical drugs into three toxicity grades; Fuzi was listed in the “inferior” grade, establishing its attribute of being a “poisonous drug.” During the Three Kingdoms period, *Pharmaceutical Records (Yao Lu)* and *Wu Pu’s Materia Medica (Wu Pu Bencao)* further defined its toxicity–efficacy correlation with descriptions such as “bitter, very warm, and toxic” (苦, 大温, 有毒). Literature from the Southern and Northern Dynasties began focusing on toxicity control strategies. For instance, *A Collection of Simple Prescriptions* (*Xiao Pin Fang*) proposed reducing toxicity by crushing Fuzi and mixing it with bitter wine for processing, pioneering the concept of processing for detoxification (*Paozhi Jiandu*). Meanwhile, *Liu Juanzi’s Prescriptions Left by Ghosts (Liu Juanzi Gui Yi Fang)* warned that combining Fuzi with dried ginger (Gan Jiang) could cause “white water toxicity” (Bai Shui toxicity, possibly referring to edema or electrolyte imbalance), reflecting an early awareness of drug interactions.

Records from the Southern Song Dynasty mark the beginning of systematic toxicological studies. Multiple classics explicitly identified its core characteristic as “acrid, hot, and highly toxic” (辛热, 有大毒) and detailed symptoms of poisoning, including headache, mucosal bleeding (“chapped lips with blood flow,” 唇裂血流), and jaundice (“body turns yellow,” 身发黄), as acute reactions. Importantly, *Essentials for Applying Drugs (Yong Yao Xin Fa)* proposed a standardized approach for “processing to reduce toxicity” using heat or wine-washing treatment. The principle, i.e., the effective destruction of highly toxic metabolites (DDAs) in Fuzi, is still validated by modern pharmacology studies. Concurrently, *Longevity and Preservation of Vitality (Shou Shi Bao Yuan)* documented a protocol using sesame oil to counteract Fuzi poisoning (“sesame oil counteracts Fuzi toxicity,” 香油解附子毒), which shows conceptual relevance to modern findings that lipid substances can antagonize the neurotoxicity of AC alkaloids.

Literature from the Ming Dynasty achieved breakthroughs in understanding the toxic mechanisms and clinical contraindications. Researchers not only systematically recorded the typical toxic manifestations induced by Fuzi, such as skin erythema (“body and eyes turn red,” 身目发红) and central nervous system excitation (“red eyes and mania,” 目赤狂躁), but also discovered its potential to cause circulatory collapse (“the whole body turns black,” 遍身皆黑) and pregnancy risks (“abortion,” 堕胎). Furthermore, *Illustrated Investigation of Plant Names and Descriptions (Zhi Wu Ming Shi Tu Kao)* drew an analogy to “arrow poison” [Jian Du, referring to the toxic latex of *Antiaris toxicaria* (J.F.Gmel.) Lesch., Moraceae] and vividly illustrated the extreme toxicity of Fuzi alkaloids, providing a historical reference for modern comparative toxicology research (Table 1).

**TABLE 1:** Historical records for toxicities of Fuzi (Aconiti Lateralis Radix Praeparata).

Dynasty	Details	Original record (in Chinese)	Origin (English translation + *Pinyin*)
Han	Classified as “inferior grade” due to toxicity	附子被列入下等毒性	*Shennong’s Classic of Materia Medica* (*Shennong Bencao Jing*)
Three Kingdoms	Described as “bitter, very warm, toxic”	苦, 大温, 有毒	*Pharmaceutical Records* (*Yao Lu*)
Three Kingdoms	Described as “toxic”	有毒	*Wu Pu’s Materia Medica* (*Wu Pu Bencao*)
Southern and Northern	Toxicity from combined use with dried ginger causes “white water” syndrome	有白水者, 是附子, 干姜毒	*Liu Juanzi’s Prescriptions Left by Ghosts* (*Liu Juanzi Gui Yi Fang*)
Southern and Northern	Processing method: crush two pieces and mix with strong vinegar	附子二枚, 捣为屑, 以淳苦酒和	*A Collection of Simple Prescriptions* (*Xiao Pin Fang*)
Southern Song	Described as “very hot, toxic”	大热附子, 有毒	*Requesting Medical Advice from Famous Doctors of All Dynasties* (*Lidai Mingyi Meng Qiu*)
Southern Song	Described as “acrid, hot, highly toxic”	味辛, 性热, 有大毒	*Lingnan Hygiene Recipes* (*Lingnan Weisheng Fang*)
Southern Song	Requires processing (e.g., roasting) to control toxicity (referring to Chuanwu and Fuzi)	川乌, 附子须炮, 以制毒也	*Essentials for Applying Drugs* (*Yong Yao Xin Fa*)
Southern Song	Described as “acrid, sweet; pickled ones very salty; extremely hot, Yang within Yang, toxic”	附子百二六 气味辛甘, 腌者大咸, 性大热, 阳中之阳也。有毒	*Orthodox Materia Medica* (*Bencao Zheng*)
Southern Song	Overdose causes head heaviness (“heavy as a peck”), chapped lips with bleeding, or jaundice	服附子多而觉头重如斗, 唇裂血流或身发黄	*Quick Guide to Recognizing Diseases* (*Shi Bing Jie Fa*)
Southern Song	Described as “acrid, sweet; warm, extremely hot, highly toxic”	附子, 味辛甘, 气温大热, 有大毒	*Complete Book for Sore and Ulcer Experiences* (*Chuangyang Jingyan Quanshu*)
Southern Song	Alcohol overdose causes head swelling, chapped lips with bleeding, or internal heat signs	凡服附子酒多, 而觉头肿唇裂血流, 或见内热诸证	*Complete Works of Jingyue* (*Jingyue Quanshu*)
Southern Song	Described as “acrid, extremely hot, toxic”	附子:味辛, 大热, 有毒。	*Selected Records of Cold Damage* (*Shanghan Xuanlu*)
Southern Song	Fuzi toxicity causes persistent vomiting; sesame oil counteracts it immediately	附子之毒, 呕吐不止, 以香油灌下立解	*Longevity and Preservation of Vitality* (*Shou Shi Bao Yuan*)
Ming	Overdose causes redness of the body and eyes	服附子过多身目发红者	*Wentang Collected Empirical Recipes* (*Wentang Jiyan Fang*)
Ming	Toxicity symptoms: head swollen “like a peck,” chapped lips with bleeding; poisoning causes red eyes and mania	附子毒头肿如斗, 唇裂血流 中附子毒目赤狂躁	*Essentials for Acute Syndromes* (*Baozheng Zhiyao*)
Ming	Redness of body/eyes after ingestion indicates poisoning	服附子后, 身目红者, 乃附毒之过	*Mirror of Medicines* (*Yao Jian*)
Ming	Described as “acrid, sweet; extremely hot, highly toxic”	味辛, 甘, 性大热, 有大毒	*Secretly Transmitted Rhyming Formulary of Materia Medica Properties* (*Michuan Yinzhi Bencao Dacheng Yaoxing Fu*)
Ming	Described as “acrid, hot, highly toxic”	味辛性热, 有大毒	*Mirror of Medicines* (*Yao Jian*)
Ming	Described as “acrid, hot, toxic”	附子辛热, 有毒	*Penetrating Materia Medica* (*Bencao Tongxuan*)
Ming	Redness of body/eyes after use indicates poisoning	用附子后, 身目红者, 乃附子之过	*Scarlet Water Mysterious Pearl* (*Chishui Xuanzhu*)
Ming	Fuzi, Chuanwu, and Caowu described as “acrid, sweet, extremely hot, toxic”	附子, 乌, 雄, 味并辛甘大热, 有毒。	*Rhyming Formulary of the Green Capsule* (*Qingnang Yaoxing Fu*)
Ming	Redness of body/eyes after use indicates poisoning	用附子后身目红者, 乃附毒之过	*Annotations and Clarifications of the Six Books on Cold Damage* (*Shanghan Liushu Zuanyao Bianyi*)
Ming	Described as “acrid, sweet; warm, extremely hot, highly toxic”	味辛, 甘, 气温, 大热。有大毒	*Essentials of Materia Medica* (*Bencao Jiyao*)
Ming	Described as “acrid, sweet; warm, extremely hot, highly toxic”	附子味辛, 甘, 气温, 大热, 有大毒。	*Complete Book for Protecting Infants* (*Bao Chi Quanshu*)
Ming	Redness of body after use indicates poisoning	用附子后, 身自红者, 乃附毒之过	*New Knowledge in Medicine, Complete Book* (*Yixue Xinzhi Quanshu*)
Ming	Overdose causes head heaviness (“heavy as a peck”) and chapped lips with bleeding	服附子过多, 而觉头重如斗, 唇裂血流	*Miraculous Remedies* (*Qixiao Liangfang*)
Ming	Chuanwu/Fuzi poisoning causes agitation and restlessness	中川乌, 附子毒者, 必烦闷	*Spring in the Apricot Garden* (*Xingyuan Shengchun*)
Ming	Described as “extremely acrid, extremely hot, highly toxic”	大辛, 大热, 大毒	*Mysterious Pearl for Smallpox Recorded by Ren Duan* (*Renduan Lu Douzhen Xuanzhu*)
Ming	Toxicity causes agitation and restlessness; severe cases include head tingling, whole-body blackening, and critical/fatal outcomes	附子毒, 心烦躁闷, 甚则头岑岑然, 遍身皆黑, 势危必死	*Introduction to Medicine* (*Yixue Rumen*)
Ming	Described as “warm, extremely hot; acrid, sweet, highly toxic”	性温, 大热, 味辛甘, 有大毒	*Differentiation and Discussion of Cold Damage* (*Shanghan Shanglun Biansi*)
Ming	Poisoning symptoms include head swelling, chapped lips, and bleeding sores	中附子毒, 头肿唇裂, 血流疮疽	*Luo’s Syncretic Medical Mirror* (*Luoshi Huiyue Yijing*)
Ming	Described as “extremely acrid, extremely hot; slightly bitter/sweet, highly toxic”	气味大辛大热, 微兼甘苦而有大毒	*Hook and Thread of Materia Medica* (*Bencao Shugou Yuan*)
Ming	Described as “fierce, dry, hot; highly toxic”; misuse causes abscesses, vision/dental issues, gastrointestinal damage, and reckless bleeding	性烈燥热, 有大毒, 误服发腰疽痈毒, 目昏齿烂, 伤损肠胃, 血热妄行, 受害不浅	*Great Compendium of Obstetrics* (*Taichan Dafa*)
Ming	Described as “acrid, hot, highly toxic”	辛, 热, 大毒	*Orthodox Center of Medical Classics* (*Yijing Yunzhong*)
Ming	Wild varieties (Shewang) used as lethal arrow poison (“seals throat upon blood contact”); smaller ones are fiercely toxic	其野生者为射罔, 制为膏以淬箭, 所中立毙, 俗谓见血封喉 小者毒烈	*Illustrated Investigation of Plant Names and Descriptions* (*Zhiwu Mingshi Tukao*)
Ming	Described as “highly toxic”	有大毒	*Medical Differentiation* (*Yixue Bianzheng*)
Ming	Described as “acrid, sweet, toxic, extremely hot”	附子 味辛甘, 有毒而大热	*New Book on Smallpox Vaccination* (*Zhongdou Xinshu*)
Ming	Severe poisoning causes agitation, head tingling, whole-body blackening, and death	心烦躁闷, 甚则头岑岑然, 遍身皆黑而死	*Complete Book of External Medicine* (*Yangyi Daquan*)
Ming	Alcohol overdose causes head heaviness (“heavy as a peck”) and chapped lips with bleeding	服附子酒多, 头重如斗, 唇裂血流	*Siyuan Hall Classified Prescriptions* (*Siyuantang Leifang Daquan*)
Ming	Symptoms: agitation and restlessness; severe cases: whole-body blackening	心烦躁闷, 甚则遍身皆黑	*Good Prescriptions for Syndrome Differentiation* (*Bianzheng Liangfang*)
Ming	Described as “toxic; many avoid its use”	附子有毒, 多不敢用	*Complete Works of Jingyue, Expanded* (*Jingyue Quanshu Fahui*)
Ming	Described as “acrid, extremely hot, pure Yang, toxic”	味辛大热, 纯阳有毒	*Seeking Truth in Materia Medica* (*Bencao Qiuzhen*)
Ming	Targets life gate meridian; described as “acrid, extremely hot, pure Yang, toxic”	专入命门 味辛大热纯阳, 有毒	*Collected Annotations on Materia Medica* (*Bencao Huizuan*)
Ming	Described as “acrid, sweet, toxic, extremely hot”	辛, 甘, 有毒, 大热	*Easy Guide to Compendium of Materia Medica* (*Bencao Gangmu Yizhilu*)
Ming	Overdose causes abscesses/sores	服附子过多, 必生疮毒	*Shangyou Hall Medical Cases* (*Shangyou Tang Yi’an*)
Ming	Symptoms: severe itching, deep/thready pulse, and slightly larger on heavy pressure	浑身痒甚, 脉沉涩, 重取稍大	*Classified Compilation of Medical Notes* (*Yichao Leibian*)
Ming	Symptoms: head swelling, cracked face, bleeding, or internal heat signs	头肿, 面裂, 血流, 或见内热诸证	*Complete Book of External Medicine Syndromes and Treatments* (*Waike Zhengzhi Quanshu*)
Unknown	Severe poisoning symptoms: agitation, head tingling, whole-body blackening, and certain death	心烦闷, 头岑岑然, 遍身皆黑, 必死	*Medical Fundamentals* (*Yizong Sunyi*)
Unknown	Described as “acrid, warm, extremely hot, toxic”	附子辛温, 大热有毒	*Guide to Researching Medicine* (*Yanyao Zhinan*)

### 3.3 Current research progress on Fuzi toxicity

#### 3.3.1 Acute toxicity and lethality

The lethal effects of Fuzi and its principal toxic metabolites, namely, the DDAs AC, MA, and HA, have been investigated in several rodent models. It has been reported that the LD_50_ values of AC in mice are approximately 1.0 mg/kg–1.8 mg/kg determined by intragastric (i.g.) administration and 0.308 mg/kg by intraperitoneal (i.p.) injection (Yang et al., 2018; Lu et al., 2022), whereas in rats, an LD_50_ of 0.064 mg/kg was determined by intravenous (i.v.) injection (Gutiérrez et al., 1987). The corresponding LD_50_ values of MA and HA in mice by i.g. administration were reported as approximately 1.9 mg/kg and 5.8 mg/kg, respectively (Yang et al., 2018). In contrast, the MDAs, which are hydrolysis products of DDAs formed during processing, exhibit markedly lower acute toxicity, with LD_50_ values of 1.50 g/kg for benzoylaconine (BAC), 0.81 g/kg for benzoylmesaconine (BMA), and 0.83 g/kg for benzoylhypaconine (BHA) in mice by i.g. administration, corresponding to an approximately 10^3^-fold reduction in toxicity compared with the parent DDAs. The minimum lethal dose of AC in humans via oral administration has been estimated at approximately 1 mg–2 mg (Yang et al., 2018; Li et al., 2025).

Using crude Fuzi preparations, Sun et al. compared the acute toxicity of raw Fuzi suspension (FZ-0) and Fuzi decocted for 60 min (FZ-60) or 120 min (FZ-120) in ICR mice at an equivalent crude drug dose of 130 g/kg (i.g.) and reported that FZ-0 caused 100% mortality within the first day, FZ-60 caused 33.3%–55.6% mortality by day 7, and FZ-120 caused no deaths during the observation period (Sun et al., 2018). However, histopathological examination revealed that even FZ-120 induced a significant decrease in the liver index and cytoplasmic and fatty degeneration of hepatocytes, indicating that decoction can effectively abolish lethality but does not completely eliminate organ toxicity (Sun et al., 2018).

The safety margins of processed Fuzi products have also been examined. A recent evaluation of HSP and PFP aqueous extracts in rats found no observed adverse effect levels (NOAELs) of 7.5 g/kg and 15 g/kg, respectively, for single-dose administration and 15 g/kg for 14-day repeated dosing, with no lethality observed at any tested dose (Ji, et al., 2023). Notably, the pathological states may markedly alter the acute toxicity of Fuzi: in a kidney yang-deficiency mouse model, a single high dose of Fuzi aqueous extract (138 g/kg, i.g.) induced 80% mortality, whereas the same dose caused no deaths in normal mice, and this was accompanied by a 20% reduction in hepatic CYP3A4 activity (Jiang, et al., 2024).

In a zebrafish model, decoctions prepared from Fuzi produced in five different regions of China were compared, and significant regional differences were observed pertaining to acute toxicity, with LC_50_ values ranging from 4.223 mg/mL for Jiangyou-Fuzi (highest toxicity) to 29.62 mg/mL for Ludian-Fuzi (lowest toxicity), which may be related to differences in the DDA content among these geo-authentic and non-geo-authentic materials (Pan et al., 2024).

Taken together, these data indicate that acute toxicity of Fuzi is driven primarily by DDAs, is greatly reduced by hydrolysis to MDAs through adequate decoction or processing, and can be significantly influenced by both the pathological state of the recipient and the geographical origin of the raw material.

#### 3.3.2 Cardiotoxicity

Cardiotoxicity is the most comprehensively investigated and clinically significant toxic effect associated with Fuzi and its DDAs, particularly AC. Preclinical evidence indicates that AC can induce marked electrophysiological abnormalities and myocardial injury phenotypes, and adverse cardiac events constitute a major feature of AC poisoning (Jiang et al., 2022).

Mechanistically, potassium-channel blockade has been proposed as one of the important pro-arrhythmic pathways. Li et al. analyzed the effects of AC on the HERG and Kv1.5 channels expressed in *Xenopus laevis* oocytes and reported that AC inhibited Kv1.5 and HERG currents in a concentration-dependent manner, with IC_50_ values of 0.796 ± 0.123 μM and 1.801 ± 0.332 μM, respectively; the voltage-/time-dependent features were consistent with preferential binding to the open state (Li et al., 2010).

In addition to K^+^ channel inhibition, Ca^2+^ signaling dysregulation has also been repeatedly implicated in AC-induced cardiotoxicity. In zebrafish embryos, exposure to 2.0 and 8.0 μM AC decreased the heart rate and inhibited ventricular/atrial contraction in a dose- and time-dependent manner, and transcriptomic analysis indicated the involvement of Ca^2+^ signaling pathways. Consistently, in H9c2 cells treated with the half-maximal inhibitory concentration of AC for 30 min, intracellular Ca^2+^ oscillation and apoptosis-related changes were observed, including decreased TnT and Bcl-2 protein expression and increased caspase-3 and Bax protein expression (Li et al., 2020).

A systematic review and meta-analysis conducted in 2022 synthesized preclinical evidence on AC-induced cardiotoxicity and concluded that AC can disturb Na^+^/Ca^2+^/K^+^ currents and trigger mitochondrial dysfunction via apoptosis/autophagy pathways and that NLRP3 inflammasome over-activation may further exacerbate myocardial injury. Notably, this meta-analysis described explicit database sources, a detailed search string, and predefined inclusion/exclusion criteria, illustrating a methodology that can be mirrored when describing the Materials and Methods of the present review (Jiang et al., 2022).

Taken together, the current evidence supports cardiotoxicity as a key target-organ toxicity of AC/Fuzi, backed by convergent findings from electrophysiology, zebrafish, and mammalian cardiomyocyte models. However, many mechanistic studies rely on simplified model systems (such as *Xenopus* oocytes or immortalized H9c2 cells) and exposure settings that may not reflect clinically relevant internal doses, which should be considered when extrapolating these findings to real-world safety evaluation of processed Fuzi products (Li et al., 2010).

#### 3.3.3 Hepatotoxicity

The hepatotoxicity of Fuzi and its principal metabolites has been documented in both *in vivo* and *in vitro* studies. Chen et al. reported that MA administration to SD rats (i.g., 10 mL/kg) induced significant elevations of serum ALT and AST levels, with hepatocyte necrosis and inflammatory infiltration confirmed by histopathology; metabolomics combined with network toxicology analysis revealed dysregulation of the HIF-1, MAPK, PI3K-Akt, and FoxO signaling pathways, along with upregulation of HMOX1, IL-2, and caspase-3 pathways (Chen et al., 2022). Similarly, Yang et al. reported dose-dependent hepatotoxicity caused by Fuzi extract in SD rats (i.g.), with a dose of 5 g/kg elevating serum AST and 10 g/kg inducing liver edema and necrosis along with significant increases in both ALT and AST levels (Yang et al., 2018).

In the subacute setting, Lu et al. demonstrated that AC in the middle- and high-dose groups (0.28 and 0.56 μmol/L, i.g., 30 days) increased ALT and AST levels progressively with a clear time–dose relationship, accompanied by histopathological changes including disorganized hepatic cords, granular degeneration, and cytoplasmic vacuolization (Lu et al., 2022). Furthermore, Sun et al. observed that even FZ-120 (120-min decocted Fuzi) at a dose of 130 g/kg (i.g.) produced subclinical hepatotoxicity in mice—no biochemical changes at the serum level, yet a decreased liver index and fatty degeneration were evident on histopathology—indicating that processing reduces but does not completely eliminate the hepatotoxic potential (Sun et al., 2018).

At the computational and cellular levels, a network toxicology study using the toxicological equivalent concentration (TEC) framework identified 22 metabolites from HSP as potentially hepatotoxic, with AKT1, IL-2, F2, GSR, and EGFR as the key targets mediating oxidative stress and apoptosis via Th17 differentiation and glutathione metabolism pathways (Zhang K. et al., 2020). More recently, a 2025 zebrafish study demonstrated that MA induced hepatocyte apoptosis through ROS accumulation and activation of the unfolded protein response (UPR)/endoplasmic reticulum (ER) stress pathway, along with upregulation of GRP78, CHOP, and caspase-12 (Tian et al., 2025).

Taken together, convergent *in vivo* and *in vitro* evidence confirms hepatotoxicity as a significant target-organ risk induced by Fuzi, particularly at supra-therapeutic doses and with prolonged exposure. However, the network toxicology study (Zhang K, et al., 2020) relied entirely on computational prediction with only limited *in vitro* validation in HepaRG cells, and several *in vivo* studies did not fully characterize the chemical composition of the Fuzi preparations used, which limits cross-study comparability and reproducibility (see Table 2).

**TABLE 2:** Summary of the modern toxicological studies on Fuzi (*Aconitum carmichaelii* Debeaux) and its principal toxic metabolites.

Animal/Cells	Extracts/Components	Administration route	Regimen	Detail effects	Controls	Ref
Acute toxicity and lethality						
ICR mice (n = 10/group)	Aconitine (AC, purity >98%)	i.g.	Single dose and multiple dose levels	LD_50_ = 1.0 mg/kg–1.8 mg/kg; 0.2 mg/kg → abnormal pulsation; 1.0 mg/kg → arrhythmia, 14% mortality	Vehicle (saline)	Yang, et al. (2018)
ICR mice	Mesaconitine (MA, purity >98%)	i.g.	Single dose	LD_50_ = 1.9 mg/kg	Vehicle	Yang, et al. (2018)
ICR mice	Hypaconitine (HA, purity >98%)	i.g.	Single dose	LD_50_ = 5.8 mg/kg	Vehicle	Yang, et al. (2018)
ICR mice	BAC/BMA/BHA (MDAs)	i.g.	Single dose	LD_50_: BAC 1.50 g/kg, BMA 0.81 g/kg, and BHA 0.83 g/kg (∼1,000-fold less toxic than DDAs)	Vehicle	Yang, et al. (2018)
Wistar rats	AC (purity >98%)	i.v.	Single dose	LD_50_ = 63.5 μg/kg (0.064 mg/kg)	Vehicle	Gutiérrez, et al. (1987)
ICR mice (n = 10/group)	FZ-0 (raw Fuzi suspension)	i.g.	130 g/kg × 7 days	100% mortality at day 1	Blank control	Sun, et al. (2018)
ICR mice (n = 10/group)	FZ-60 (60-min decocted Fuzi)	i.g.	130 g/kg × 7 days	33.3%–55.6% mortality by day 7; DDA content ↓ with decoction time	Blank control	Sun, et al. (2018)
ICR mice (n = 10/group)	FZ-120 (120-min decocted Fuzi)	i.g.	130 g/kg × 7 days	No deaths; histopathological liver damage detected	Blank control	Sun, et al. (2018)
SD rats (n = 10/group)	Heishunpian (HSP) aqueous extract	i.g.	Single dose + 2-week repeated dose	NOAEL: 7.5 g/kg (bolus), 15 g/kg (2-week); no lethality at tested doses	Vehicle (water)	Ji, et al. (2023)
SD rats (n = 10/group)	Paofupian (PFP) aqueous extract	i.g.	Single dose + 2-week repeated dose	NOAEL: 15 g/kg; no lethality	Vehicle (water)	Ji, et al. (2023)
KM mice	Fuzi aqueous extract	i.g.	138 g/kg single dose	0% mortality in normal mice; 80% mortality in the kidney yang deficiency model (↓CYP3A4 activity)	Normal + model control	Jiang, et al. (2024)
Zebrafish larvae (6 dpf)	Fuzi decoctions from five Fuzi-producing regions	Immersion	LC_50_ determination, 24 h	LC_50_ (mg/mL): Ludian 29.62, Anguo 4.887, Chenggu 18.42, Butuo 4.223, and Jiangyou highest toxicity	Blank water	Pan, et al. (2024)
Cardiotoxicity						
H9c2 cells	AC (purity ≥98%)	Cell culture	50 μM–200 μM, 24 h	Viability: 58% ± 2.91% (100 μM), 10% ± 0.5% (200 μM); ↓PGC-1α, ↓ΔΨm, ↑Bax/Bcl-2, ↑caspase-3, ↑cytochrome C release	Untreated cells	Gao, et al. (2018)
H9c2 cells	AC	Cell culture	0.5 and 1.0 μmol/L, 24 h	TNFα↑, NLRP3↑; BNIP3-dependent mitophagy inhibited → inflammation + apoptosis; BNIP3 overexpression reversed damage	Untreated + BNIP3 overexpression	Peng, et al. (2020)
HEK293 cells (*HERG/Kv1.5* transfected)	AC (purity >98%)	Patch-clamp	IC_50_ determination	IC_50_ of HERG block: 1.801 μM; IC_50_ of Kv1.5 block: 0.796 μM; open-state block mechanism	Vehicle	Li, et al. (2010)
hiPSC-CMs	AC	Cell culture	0.25 μM–3.0 μM	0.25 μM: beating rate ↑3.7×; 3.0 μM: ↑7.3×; L-type Ca^2+^ channel	Vehicle (0.1% DMSO)	Zhang F, et al. (2018)
ICR mice (n = 10/group)	AC (purity >98%)	i.g.	0.146 mg/kg/d × 7 days	VT, ↑CK/AST/LDH; hyperchromatic nuclei, and condensed cytoplasm in myocardial tissue	Vehicle	Yang, et al. (2018)
Beagle dogs (n = 6/group)	HA (purity >98%)	i.g.	Single dose: 0.05, 0.15, and 0.45 mg/kg	Dose-dependent QT prolongation; Cmax = 1.53 ng/mL at 0.05 mg/kg	Vehicle	Yang, et al. (2018)
SD rats (n = 10/group)	Fuzi extract (DDAs quantified)	i.g.	2-, 5-, and 10-g/kg single dose	2 g/kg: mild edema; 5 g/kg: ↑LDH; 10 g/kg: ↑CK/LDH/AST, nuclear variation	Vehicle (water)	Yang, et al. (2018)
— (meta-analysis)	AC	—	13 preclinical studies pooled	Systematic review: Na^+^ channel persistent activation, Ca^2+^ overload, mitochondrial dysfunction, and NLRP3 inflammasome are the core cardiotoxicity mechanisms	—	Jiang, et al. (2022)
Human (clinical case)	AC (blood level: 65.6 μg/mL)	Oral	Accidental ingestion	Malignant VT → cardiogenic shock → heart transplant; acute myocardial necrosis confirmed by pathology	—	Liao et al. (2022)
Zebrafish embryos (48 hpf)	AC	Immersion	Multiple concentrations	Ca^2+^ signaling disruption → cardiac dysfunction; ↑apoptosis in the heart region	Blank water	Li, et al. (2020)
Hepatotoxicity						
SD rats (n = 6/group)	MA (purity >98%)	i.g.	10 mL/kg, observed 14 days	↑ALT/AST; hepatocyte necrosis, inflammatory infiltration; HIF-1/MAPK/PI3K-Akt/FoxO pathways	Vehicle (saline)	Chen, et al. (2022)
Wistar rats (n = 10/group)	HSP aqueous extract	i.g.	20 and 40 g/kg/d × 20 days	↑ALT/AST (both groups); lipid peroxidation product accumulation	Low-dose group as reference	Li, et al. (2025)
ICR mice (n = 10/group)	FZ-120 (2 h-decocted Fuzi)	i.g.	130 g/kg × 7 days	No serum biochemical changes; but ↓liver index, cytoplasmic + fatty degeneration (histopathology)	Blank control	Sun, et al. (2018)
SD rats	Fuzi extract (DDAs quantified)	i.g.	5- and 10-g/kg single dose	5 g/kg: ↑AST; 10 g/kg: liver edema/necrosis, ↑ALT/AST	Vehicle	Yang, et al. (2018)
— (network toxicology + *in vitro*)	22 toxic metabolites from HSP	Computational + HepaRG cells	TEC framework	Targets: AKT1, IL2, F2, GSR, and EGFR Pathways: Th17/Jak-STAT/glutathione → oxidative stress + apoptosis	—	Zhang K, et al. (2020)
Zebrafish larvae (5 dpf)	MA	Immersion	Multiple concentrations, 72 h	ROS↑ → UPR/ER stress activation → hepatocyte apoptosis; GRP78↑, CHOP↑, caspase-12↑	Blank water	Tian, et al. (2025)
Nephrotoxicity						
SD rats (n = 10/group)	Fuzi extract (DDAs quantified)	i.g.	5- and 10-g/kg single dose	5 g/kg: scattered lymphocytes; 10 g/kg: renal atrophy, ↑urea	Vehicle	Yang, et al. (2018)
SD rats	Clinically relevant dose HSP/PFP	i.g.	Single +15-day	Six alkaloids quantified (UPLC-MS/MS): kidney was the major accumulation organ (1.8–11.7× blood)	Vehicle	Ji, et al. (2019)
— (network toxicology)	Fuzi metabolites	Computational + MS screening	—	Multiple nephrotoxic metabolites identified; multi-target, multi-pathway	—	Yin, et al. (2024)
Neurotoxicity						
Human (n = 41 cases)	AC from *Aconitum* (clinical poisoning)	Oral	Various doses	Paresthesia (most common), tremors, and consciousness disturbance; neurological symptoms > cardiac symptoms in prevalence; alcohol-processed AC worsened outcomes	—	Chung et al. (2021)
SD rats (n = 8/group) + PC12 cells	AC (purity >98%)	i.g. (*in vivo*); cell culture (*in vitro*)	0.5, 1.5, and 2.5 mg/kg (rats); 5 μM–80 μM (PC12)	↑BBB permeability; PC12 proliferation inhibition + apoptosis; ER stress (GRP78↑); dose-dependent	Vehicle (saline); untreated cells	Zhang X, et al. (2022)
HT22 hippocampal cells	AC	Cell culture (chip-MS)	Multiple concentrations	Excitotoxicity: Glu/Asp↑ → Ca^2+^ overload → oxidative stress → apoptosis; LDH↑	Untreated cells	Zhang Y, et al. (2023)
Zebrafish larvae (6 dpf)	Fuzi decoctions (five regions)	Immersion	Sub-lethal conc., 24 h	Jiangyou-Fuzi highest neurotoxicity; ↓locomotion; GABA↑, Glu↓, ACh↓; RNA-Seq: six key genes; neurotrophin/ErbB/cGMP-PKG/p53 pathways	Blank water	Pan, et al. (2024)
Zebrafish embryos	AC	Immersion	Sub-lethal concentration	Disrupted serotonin (5-HT) neurotransmission	Blank water	Chen, et al. (2021)
PC12 cells + ICR mice	Crude *Aconitum* extract	Cell culture; i.p	*in vitro* + *in vivo*	Dopamine release↑; ROS↑, SOD↓, GPx↓; dose-dependent neuronal apoptosis	Untreated; vehicle	Zhao, et al. (2010)
Developmental toxicity and embryotoxicity						
Zebrafish embryos (AB strain, 4–96 hpf)	AC (purity ≥98%)	Immersion	LC_1_ = 7.27 μM, LC_10_ = 8.23 μM; 96 h	Pericardial edema, yolk retention, swim bladder deficiency, brain deficiency, and curved body; ↑ROS, ↓T-SOD; Nrf2/HO-1↓, JNK↑, caspase-3/9↑, Bax↑	Blank E3 medium	Xia, et al. (2021)
Rat whole embryo culture (WEC, GD 9.5)	AC/MA/HA + BAC/aconine	WEC system	10, 5, 2.5, and 1.25 μM, 48 h	AC most embryotoxic; DDAs→malformation (neural tube and heart); p53/p21↑, CDK2/NPAT↓; BAC/aconine significantly less toxic	Vehicle (DMSO)	Feng, et al. (2025)
Rat embryos (GD 9.5, WEC)	AC (purity >99%)	WEC system	0.5 μg/mL–5.0 μg/mL, 48 h	NOAEL = 1 μg/mL; 2.5 μg/mL: ↓crown–rump length; 5 μg/mL: cardiac + brain malformation; concentration-dependent	Vehicle (DMSO)	Xiao, et al. (2007)
Reproductive toxicity and genotoxicity						
Male ICR mice (n = 10/group)	Fuzi (raw) aqueous extract	i.g.	5.85 g/kg/d × 14 days	↓Testis weight, ↓sperm count/viability, ↑sperm deformity; ↓testosterone; ↓SOD/GSH/CAT, ↑MDA; ↑DNA damage (comet assay); ↑micronucleus rate	Vehicle (water)	Zhang K, et al. (2021)
Male ICR mice (n = 10/group)	HSP/DFP aqueous extracts	i.g.	5.85 g/kg/d × 14 days	Reproductive + genotoxicity significantly reduced vs. raw Fuzi; HSP and DFP showed lowest toxicity	Vehicle + raw Fuzi group	Zhang K, et al. (2021)
Gastrointestinal toxicity						
Human (clinical review)	AC/MA/HA	Oral	Poisoning cases	Nausea, vomiting (earliest symptoms), diarrhea, abdominal pain; AC/MA/HA → ileum contraction via ACh release from postganglionic cholinergic nerves	—	Chan (2009)
Human (n = 8 cases)	AC (Chinese patent medicine and misuse)	Oral	Accidental ingestion	All eight patients: GI symptoms (nausea/vomiting/abdominal pain) + arrhythmia; one cardiac arrest → ECMO; all survived	—	Shang, et al. (2025)
Subacute and chronic toxicity						
ICR mice	AC (purity >98%)	i.g.	Low/mid/high dose × 10–30 days	↓Body weight (high dose); organ coefficient changes (liver, spleen, and brain); hematological alterations	Vehicle (saline)	Lu et al. (2022)
ICR mice	AC (purity >98%)	i.g.	0.6 mg/kg/d × 14 days → 1.8 mg/kg bolus day 15	Adaptive tolerance: prior low-dose exposure reduced mortality from subsequent lethal bolus; AC Cmax in tissues ↓ over days 10–22	Naïve mice receiving the same bolus	Yang, et al. (2018)
SD rats	HSP/PFP (clinical dose)	i.g.	Single +15-day repeated	Six alkaloids in plasma/organs by UPLC-MS/MS; liver > kidney > heart > brain distribution; accumulation with repeated dosing	Vehicle	Ji, et al. (2019)

#### 3.3.4 Nephrotoxicity

Nephrotoxic effects of Fuzi have been observed in multiple animal studies. Yang et al. reported that Fuzi extract at 5 g/kg (i.g., single dose) induced scattered lymphocytic infiltration in the renal parenchyma of SD rats, while the 10-g/kg dose caused renal atrophy accompanied by elevated serum urea nitrogen (Yang, et al., 2018). In the subacute model, Lu et al. found that AC at 0.28 and 0.56 μmol/L (i.g., 10–30 days) progressively elevated serum BUN and creatinine (CRE) levels with a clear time–dose relationship; histopathologically, flocular degeneration of renal tubular epithelial cells and interstitial hyperemia and hemorrhage were observed in the middle- and high-dose groups, with tubular casts appearing in the high-dose group by day 30 (Lu et al., 2022).

Toxicokinetic tissue distribution studies using UPLC-MS/MS quantification revealed that Fuzi alkaloids are markedly accumulated in the kidney, with tissue-to-blood ratios of 1.8–11.7-fold for the six measured alkaloids after both single-dose and 15-day repeated administration in SD rats, and the accumulation increased with repeated dosing (Ji et al., 2019). A recent study using UHPLC-Q-Exactive-Orbitrap MS screening identified that 81 Fuzi metabolites entered the blood, of which mesaconine, BAC, AC, and HA were flagged as potential nephrotoxic substances through network toxicology studies, with AKT1, IL-2, and F2 predicted as the key targets (Yin, et al., 2024) (see Table 2).

Despite these findings, the nephrotoxicity evidence base remains limited compared to that of other organ systems. The available *in vivo* data (Yang et al., 2018; Lu et al., 2022) did not employ modern, kidney-specific biomarkers such as KIM-1, NGAL, or cystatin C, which would more sensitively detect early tubular damage; and no dedicated *in vitro* studies using renal tubular cell lines (e.g., HK-2 or NRK-52E) have been conducted, leaving the cellular mechanisms of Fuzi-induced nephrotoxicity largely uncharacterized.

#### 3.3.5 Neurotoxicity

Neurotoxicity is a clinically prominent toxic feature of Fuzi and its metabolites. A retrospective analysis of 41 clinical poisoning cases revealed that neurological symptoms—including paresthesia, tremors, and impaired consciousness—were more prevalent than cardiac events; notably, patients who ingested alcohol-processed AC experienced more severe neurological outcomes, indicating that ethanol potentiates AC neurotoxicity (Chung et al., 2021). At the *in vivo* level, AC at doses of 0.5, 1.5, and 2.5 mg/kg (i.g.) induced dose-dependent increases in blood–brain barrier (BBB) permeability in SD rats. In the same study, AC at 5 μM–80 μM inhibited PC12 cell proliferation and induced apoptosis through the activation of ER stress signaling, with the upregulation of GRP78 expression, indicating that ER stress is a mechanism underlying AC neurotoxicity (Zhang X, et al., 2022).

Furthermore, AC induces excitotoxicity in HT22 hippocampal cells by increasing the extracellular release of glutamate and aspartate, leading to intracellular Ca^2+^ overload, oxidative stress, and apoptosis, as evidenced by elevated LDH release (Zhang Y, et al., 2023). Zhao et al. reported that crude *Aconitum* extract stimulated dopamine release from PC12 cells in both *in vitro* and *in vivo* settings, accompanied by decreased SOD and GPx activity and increased ROS production, resulting in dose-dependent neuronal apoptosis (Zhao et al., 2010). In addition, AC has been shown to disrupt serotonin (5-HT) neurotransmission in a zebrafish model (Chen et al., 2021).

A recent comparative study using Fuzi decoctions from five major Fuzi-producing regions of China demonstrated significant regional variation in neurotoxicity in zebrafish larvae (6 dpf): Jiangyou-Fuzi exhibited the highest neurotoxicity (lowest LC_50_), with decreased locomotion, decreased acetylcholine and glutamate levels, and increased GABA levels; RNA-seq analysis identified six key genes and four signaling pathways (neurotrophin, ErbB, cGMP-PKG, and p53) as the underlying mechanisms (Pan et al., 2024) (see Table 2).

The neurotoxicity evidence spans clinical, *in vivo* mammalian, and zebrafish data, providing a multi-level perspective. Nevertheless, several limitations should be noted: the clinical data (Chung et al., 2021) are retrospective and lack standardized neurological scoring; the regional comparison of neurotoxicity in zebrafish (Pan et al., 2024) did not quantify the DDA/MDA content in the five Fuzi decoctions, making it difficult to attribute toxicity differences to specific alkaloid profiles; and the crude extract used by Zhao et al. was not chemically characterized, precluding the attribution of neurotoxic effects to specific metabolites (Zhao et al., 2010).

#### 3.3.6 Developmental toxicity and embryotoxicity

The developmental toxicity of Fuzi has been characterized using both zebrafish embryo and rat whole-embryo culture (WEC) models. Xia et al. exposed zebrafish embryos (AB strain, 4 hpf–96 hpf) to AC and observed multiple developmental malformations above LC_10_ (8.23 μM), including pericardial edema, yolk retention, swim bladder deficiency, brain deficiency, and body curvature; mechanistically, these were associated with increased ROS, decreased T-SOD activity, downregulation of the Nrf2/HO-1 pathway, and upregulation of JNK, Bax, and caspase-3/9 levels (Xia et al., 2021).

In a WEC study, Xiao et al. established a NOAEL of 1 μg/mL for AC in rat embryos (GD 9.5, 48-h culture); a NOAEL of 2.5 μg/mL decreased the crown–rump length, and that of 5 μg/mL induced cardiac and brain malformations in a concentration-dependent manner (Xiao et al., 2007). A more recent WEC study (2025) by Feng et al. extended this work by comparing the embryotoxicity induced by three DDAs (AC, MA, and HA) and two MDAs (BAC and aconine) at a concentration of 1.25 μM–10 μM in rat embryos; AC was the most embryotoxic among them, inducing neural tube and cardiac malformations through p53/p21 pathway activation and CDK2/NPAT downregulation, leading to DNA damage and cell-cycle arrest; BAC and aconine induced significantly lower embryotoxicity, providing mechanistic insights that processing-induced DDA hydrolysis reduces developmental toxicity (Feng, et al., 2025) (see Table 2).

The WEC model studies (Xiao et al., 2007; Feng et al., 2025) are the most methodologically rigorous in this category, and they used well-defined concentrations of pure metabolites (purity stated) with appropriate vehicle controls. However, WEC is an *ex vivo* system that does not account for maternal metabolism or placental transfer in determining the actual embryonic exposure, and standard *in vivo* teratogenicity studies in pregnant rats or rabbits dosed during organogenesis—as required for regulatory safety assessment—are currently absent from the literature on Fuzi.

#### 3.3.7 Reproductive toxicity and genotoxicity

The reproductive toxicity and genotoxicity of Fuzi were systematically investigated by Zhang et al. in male ICR mice receiving aqueous extracts of raw Fuzi and its processed products (HSP, BFP, and Danfupian/DFP) at 5.85 g/kg/d (i.g.) for 14 consecutive days. Raw Fuzi significantly reduced testis weight and testicular coefficient, induced obvious histopathological changes in testicular tissue, decreased sperm count and viability, and increased the sperm deformity rate. Serum testosterone was significantly decreased, while oxidative stress markers showed increased MDA and decreased SOD, GSH, and CAT, indicating oxidative damage as a contributory mechanism. Genotoxicity was confirmed by increased DNA damage, as observed in the comet assay, and elevated micronucleus rates in the sperm, peripheral blood cells, and bone marrow cells. Importantly, processed products—particularly HSP and DFP— significantly attenuated reproductive and genotoxic effects compared with raw Fuzi, and this was associated with higher testosterone levels and reduced oxidative stress, further supporting the efficacy of traditional processing methods in detoxification (Zhang K. et al., 2021) (see Table 2).

This is currently the only systematic study addressing the reproductive toxicity and genotoxicity of Fuzi, representing a major evidence gap. The study was limited to male animals at a single dose level, and no standard regulatory genotoxicity assays (Ames test or *in vitro* chromosome aberration assay) or female reproductive toxicity data are available, which would be essential components of a comprehensive safety evaluation.

#### 3.3.8 Gastrointestinal toxicity

Gastrointestinal (GI) symptoms are among the earliest and most consistent manifestations of AC poisoning. Chan reported that nausea, vomiting, abdominal pain, and diarrhea typically precede cardiac symptoms by 30 min–60 min following oral AC ingestion, and mechanistic analysis indicated that AC, MA, and HA induce ileum contraction through acetylcholine (ACh) release from postganglionic cholinergic nerves; an overall case-fatality rate of 5.6% was reported in the reviewed cases (Chan, 2009). A 2025 clinical case series reported eight patients who accidentally ingested AC-containing Chinese patent medicines, all of whom presented with GI symptoms followed by cardiac arrhythmias; one patient experienced cardiac arrest, requiring extracorporeal membrane oxygenation (ECMO), and all eight ultimately survived with appropriate treatment (Shang et al., 2025) (see Table 2).

The currently available GI toxicity data are derived exclusively from clinical observations and case series, which, while informative, do not allow systematic dose–response characterization. No preclinical *in vitro* or *in vivo* studies specifically designed to evaluate Fuzi-induced GI mucosal injury, intestinal permeability changes, or the effects on the gut microbiome have been conducted, limiting the mechanistic understanding of this earliest-manifesting toxic endpoint.

#### 3.3.9 Subacute and chronic toxicity

The subacute toxicity profile of AC was characterized by Lu et al. in a 30-day mouse study using three dose groups (0.14, 0.28, and 0.56 μmol/L, i.g., n = 30/group, Kunming mice). The high-dose group exhibited significantly decreased body weight from day 20 onward, altered organ coefficients—including decreased liver and spleen coefficients and increased lung coefficient—and progressive hematological changes: RBC, HGB, HCT, and PLT decreased continuously in the middle- and high-dose groups from day 20 onward, while the WBC and granulocytes initially decreased and then increased, indicating biphasic immune modulation. Histopathological examination at day 30 revealed degeneration and necrosis in the heart and cerebellum, making them the most severely affected organs (Lu et al., 2022).

An interesting adaptive tolerance phenomenon was reported by Yang et al.: mice pretreated with a sub-lethal dose of AC (0.6 mg/kg/d, i.g.) for 14 days showed markedly reduced mortality when subsequently challenged with a lethal bolus (1.8 mg/kg) on day 15 compared with the naïve controls; AC tissue concentrations declined progressively from day 10 to day 22, indicating the induction of hepatic metabolic enzymes or enhanced elimination mechanisms (Yang et al., 2018). Toxicokinetic distribution studies using UPLC-MS/MS demonstrated that six Fuzi alkaloids accumulate preferentially in the following order: liver > kidney > heart > brain after both single-dose and 15-day repeated dosing in SD rats (Ji et al., 2019) (see Table 2). While the subacute study by Lu et al. (2022) provides the most systematic multi-organ assessment to date, the dosing concentrations were expressed in μmol/L rather than the conventional mg/kg, making direct interstudy comparison difficult. Chronic toxicity studies exceeding 90 days—necessary for evaluating the safety of long-term clinical Fuzi use—are entirely absent from the literature, as are carcinogenicity and chronic low-dose exposure data.

## 4 Mitigation of Fuzi toxicity through processing

### 4.1 Historical evolution of processing methods

The processing methods for Chinese botanical drugs, rooted in empirical practice and TCM theory, have evolved over millennia in China. These methods alter the properties and organoleptic characteristics of botanical drugs; they not only potentially enhance the therapeutic efficacy but also reduce or eliminate toxicity. Historically, Fuzi (*Aconitum carmichaelii* Debeaux, Ranunculaceae) processing evolved along four core technological axes—preparatory morphology modification (*Xiuzhi Fa*), water-based processing (*Shui Zhi Fa*), fire-based processing (*Huo Jiagong*), and combined fire–water techniques (*Huo Shui Jiagong*)—each reflecting distinct empirical strategies to balance the efficacy and toxicity. Since the Han Dynasty (202 BCE–220 CE), processing techniques for *Fuzi* (Aconiti Lateralis Radix Praeparata) have been documented in various classical Materia Medica texts throughout history. Numerous methods—such as washing, cutting, dry-frying, stir-frying with adjuvants, water-soaking (with or without alum), and decoction (with or without adjuvants)—have been reported in these historical texts (Table 3).

**TABLE 3:** Historical evolution of the processing categories for Fuzi (Aconiti Lateralis Radix Praeparata) across Chinese dynasties.

Dynasty	Processing category	Representative method (high-level summary)	Key detoxification principle/Technological feature	Representative classical source
Han (202 BCE–220 CE)	Preparation (*Xiuzhi Fa*)	Peeling and sectioning Fuzi into eight pieces prior to further processing	Morphological standardization to increase the surface area, facilitating subsequent leaching or heating	*Jingui Yuhan Fangle*
Eastern Han (25–220 CE)	Water-based processing (*Shui Zhi Fa*)	Periodic soaking of large roots in boys’ urine with daily fluid replacement (3 days in summer and 5 days in winter), followed by boiling in fresh urine until cooked, and then peeling and drying	Time-dependent aqueous leaching of DDAs; alkaline urine medium may promote partial hydrolysis of ester bonds	*Huatuo Shenfang*
Eastern Jin (317–420 CE)	Preparation (*Xiuzhi Fa*)	Pounding Fuzi into fine powder for direct oral administration	Pulverization to enhance bioavailability; no deliberate detoxification step, reflecting early empirical use	*Zhouhou Beiji Fang*
Southern and Northern Dynasties (420–589 CE)	Fire-based processing (*Huo Jiagong*)	Roasting over willow-wood fire until cracked; scraping off adventitious buds; removing the base and tip; splitting and burying underground overnight before drying	Direct thermal decomposition and hydrolysis of DDAs; underground burial may facilitate slow cooling and residual moisture-mediated leaching	*Leigong Paozhi Lun*
Southern and Northern Dynasties (420–589 CE)	Water-based processing (*Shui Zhi Fa*)	Thin-slicing Fuzi and then soaking in flowing stream water with black soybeans [*Glycine max* (L.) Merr., Fabaceae] for 5 days and nights, followed by draining and sun-drying	Enhanced leaching using flowing water; adjuvant synergy with soybean proteins/tannins potentially binding alkaloids	*Leigong Paozhi Lun*
Southern and Northern Dynasties (420–589 CE)	Preparation (*Xiuzhi Fa*)	Systematic morphological refinement: scraping protrusions, removing the base/tip, and standardized thin-slicing	Precision cutting to control slice uniformity, optimizing subsequent processing efficiency	*Leigong Paozhi Lun*
Northern Song (960–1127 CE)	Fire-based processing (*Huo Jiagong*)	Carbonizing Fuzi while retaining residual properties (*Shao Cun Xing*) and then grinding into powder	Controlled charring to destroy most DDAs while preserving thermostable metabolites	*Yanglao Fengqin Shu*
Northern Song (960–1127 CE)	Preparation (*Xiuzhi Fa*)	Differentiated protocol: raw use requires peeling and tip removal; processed use requires ash-fire roasting until cracked and then peeling	First standard distinguishing raw vs. processed Fuzi based on clinical application needs	*Michuan Yanke Longmu Lun*
Southern Song (1127–1279 CE)	Fire-based processing (*Huo Jiagong*)	Roasting until cracked, removing the peel and umbilicus, chopping, and then baking to dryness	Sequential thermal treatment with progressive morphological refinement; four-step procedural standardization	*Shi Bian Liang Fang*
Southern Song (1127–1279 CE)	Fire–water co-processing (*Huo Shui Jiagong*)	Three-stage process: stove-flue baking → hot boys’ urine soaking (5–7 days) → ash-roasting with “white star” endpoint testing	Combined thermal–aqueous processing; introduction of visual quality-control endpoint (*Bai Xing* disappearance)	*Xiaoer Douzhen Fanglun*; *Chen Shi Xiaoer Douzhen Fanglun*
Southern Song (1127–1279 CE)	Fire–water co-processing (*Huo Shui Jiagong*)	Seven iterative cycles of roasting and salt-water soaking, with peeling at the final stage	Repetitive thermal–aqueous cycling for exhaustive DDA removal; salt as an osmotic leaching enhancer	*Sanyin Jiyi Bingzheng Fanglun*; *Huoren Shizheng Fang Houji*
Southern Song (1127–1279 CE)	Water-based processing (*Shui Zhi Fa*)	Seven cycles of salt-water soaking followed by umbilicus removal; thin-slicing and sun-drying standardized	Iterative aqueous leaching with salt as an adjuvant; morphological standardization (thin slicing) for drying uniformity	*Leibian Zhu Shi Jiyan Yifang*; *Sheng Ji Zong Lu*
Yuan (1271–1368 CE)	Fire-based processing (*Huo Jiagong*)	Wrapping Fuzi in wet paper and roasting in ashes (*Hui Zhong Pao*) and then removing blackened peel	Indirect medium-controlled heating (wet paper + ash) to ensure uniform thermal conduction and minimize surface charring	*Zhenzhu Nang Buyi Yaoxing Fu*
Yuan (1271–1368 CE)	Water-based processing (*Shui Zhi Fa*)	Frequent boiling and soaking in boys’ urine to neutralize toxicity	Combined boiling and urine-soaking for dual thermal–chemical leaching	*Bencao Yanyi Buyi*
Yuan (1271–1368 CE)	Fire–water co-processing (*Huo Shui Jiagong*)	Pretreatment with wine-washing and sun-drying before further processing	Introduction of ethanol-containing medium (wine) for potential ester-bond solvolysis	*Yongyao Xinfa*
Ming (1368–1644 CE)	Water-based processing (*Shui Zhi Fa*)	Prolonged soaking in concentrated licorice [*Glycyrrhiza uralensis* Fisch. ex DC., Fabaceae] decoction for 2–3 days; multiple adjuvant variations including boys’ urine, ginger juice, and salt water	Adjuvant-mediated chemical conjugation (glycyrrhizic acid binding to DDAs); diversification of aqueous leaching media	*Bencao Zheng*; *Bencao Danfang*; *Bencao Huiyan*; *Leijing Tuyi*
Ming (1368–1644 CE)	Fire–water co-processing (*Huo Shui Jiagong*)	Seven cycles of salt-water soaking and roasting; boiling in boys urine followed by roasting; simmering with ginger juice and salt broth	Iterative thermal–aqueous cycling with structured adjuvant synergy (salt + ginger + urine)	*Chongke Wan Shi Jiachuan Jishi Liangfang*; *Chuangyang Jingyan Quanshu*; *Chishui Xuanzhu*
Ming (1368–1644 CE)	Fire-based processing (*Huo Jiagong*)	Gentle ash-fire roasting (*Tang Hui Zhong Pao*) until slight fissuring; standardized peel removal and slicing; salt-water wrapping before roasting	Precision heat control using flameless ash; morphological standardization with emerging quantitative parameters	*Bencao Pinhui Jingyao*; *Shanghan Xuanlu*; *Jinzai Yiyao*
Qing (1644–1912 CE)	Fire-based processing (*Huo Jiagong*)	Multistep protocol: ash roasting → scraping protrusions → tip removal → splitting → drying; adjuvant-enhanced methods including honey-roasting, vinegar-roasting, and dough-wrapped roasting (*Mian Guo Wei*)	Diversified indirect heating media (ash, dough, honey, and vinegar); medium-controlled heat conduction for uniform processing	*Michuan Yinzhi Bencao Dacheng Yaoxing Fu*; *Bencao Gangmu*; *Waike Zhengzong*; *Yaojian*
Qing (1644–1912 CE)	Water-based processing (*Shui Zhi Fa*)	Multiple soaking protocols: soaking in boys urine (7 days); flowing-water soaking with black soybeans (5 days); blanching in boiling water, followed by licorice decoction infusion; quantified-to-adjuvant ratios specified	Systematization with quantitative parameters (ratios, durations); “no tongue numbness” (*She Chang Bu Ma*) adopted as the sensory toxicity endpoint	*Cangsheng Siming*; *Yimen Mizhi*; *Bencao Tongxuan*; *Baoying Cuoyao*
Qing (1644–1912 CE)	Fire–water co-processing (*Huo Shui Jiagong*)	Nine cycles of steaming and sun-drying with boys’ urine; dough-wrapped roasting with ginger juice-steaming; boiling with salt, ginger juice, and licorice in a clay pot (seven cycles)	Iterative processing reaching maximal cycle counts (9×); integration of sensory endpoint (“no numbness”) with material-selection criteria (“lotus-petal aconite”); philosophy of “harmonizing fire and water”	*Bencao Gangmu Shiyi*; *Qi Shi Yi’an*; *Shoushi Baoyuan*; *Mujing Dacheng*; *Chishui Xuanzhu*
Qing (1644–1912 CE)	Fermentation-based processing	Vinegar fermentation: soaking Fuzi in barley-yeast vinegar for 7 days, allowing white mold growth, and then slow sun-drying (10 days); rice porridge–yeast fermentation	Introduction of microbial fermentation as a novel detoxification principle; enzymatic degradation of DDAs via microbial metabolites	*Binhu Paozhi Fa*
Republic of China (1912–1949 CE)	Preparation (*Xiuzhi Fa*)	Slicing after peeling; double blanching with boiling water to remove salt and residual toxicity; stir-frying in a copper vessel until cooked	Transition from traditional empirical methods to proto-standardized procedures; introduction of metal vessels for uniform heating	*Danfang Jinghua*
Republic of China (1912–1949 CE)	Fire–water co-processing (*Huo Shui Jiagong*)	Salt-wine stir-frying (*Yan Jiu Chao*); soaking in boys urine (3 days), followed by biological fermentation	Innovation of ethanol + salt combined processing; early integration of fermentation concepts into standard practice	*Danfang Jinghua*
Modern (Chinese Pharmacopoeia, 2020 edition)	Standardized pharmacopoeial processing	Salt-processed Fuzi (Yan Fuzi): salt-brine soaking and boiling; Heishunpian (HSP): staining with caramel coloring, steaming, and drying; Baifupian (BFP): peeling, slicing, alum-water boiling, and rinsing; Danfupian: desalting + co-processing with black soybean and licorice	Product-specific DDA limits (e.g., ≤0.020% for HSP/BFP) Quality control via HPLC/UPLC quantification of DDAs and MDAs; adjuvant-mediated detoxification (licorice and black soybean) retained from classical heritage	Chinese Pharmacopoeia (2020 ed.)

Detailed original classical descriptions, stepwise procedures, and full quotations for each entry are provided in Supplementary Table S1.

#### 4.1.1 Preparation methods *(Xiuzhi Fa)*

Preparation methods (*Xiuzhi Fa*, 修制法) constitute the foundational stage of TCM processing, primarily involving purification (cleaning), cutting/shaping, and pulverization of the raw botanical drug to meet the pharmaceutical standards before further processing or direct use. The historical evolution of these methods for Fuzi (附子) is outlined below.

Han Dynasty (202 BCE–220 CE): The earliest documented preprocessing instructions for aconite appear in the *Jingui Yuhan Fanglüe* (*Synopsis of Prescriptions of the Golden Chamber* with Jade Case), mandating peeling (“*qu pi*”, 去皮) and sectioning into eight pieces (“*po ba pian*”, 破八片). This established the necessity for morphological modification prior to medicinal use, laying the groundwork for subsequent techniques.

Eastern Jin Dynasty (317–420 CE): The *Zhouhou Beiji Fang (Handbook of Prescriptions for Emergencies)* introduced primitive pulverization, exemplifying an early integration of traditional techniques with rudimentary pharmaceutical preparation principles.

#### 4.1.2 Water-based processing (*Shui Zhi Fa*)

Water-based processing (*Shui Zhi Fa*, 水制法) uses water or other liquid adjuvants without heating to modify botanical drugs, primarily through washing, soaking, and moistening. The earliest record of its application to Fuzi (附子) is found in the *Jingui Yuhan Jing* (*Synopsis of Prescriptions of the Golden Chamber* with Jade Case), underscoring water’s critical role in ancient detoxification practices. *Hua Tuo Shen Fang (Hua Tuo’s Divine Formulas),* literature from the Eastern Han Dynasty, established a periodic soaking protocol using boys’ urine (“*tong bian*”, 童便), where large aconite roots were submerged to a depth of 3 inches (*cun*), with daily replacements for 3 days in summer or five in winter, setting a standard for time-dependent fluid interaction.

From the Wei-Jin to Yuan Dynasties, the techniques expanded. The *Bencao Yanyi Buyi (Supplement to the Augmented Materia Medica)* combined boiling with urine-soaking (“*tong bian zhu er jin zhi*”, 童便煮而浸之). The *Leigong Paozhi Lun* (Northern and Southern Dynasties) introduced soaking in east-flowing stream water (“*dong liu shui*”, 东流水) mixed with black soybeans (*heidou*, 黑豆) for 5 days and nights, pioneering the use of “living water” and adjuvant synergy.

The Song Dynasty saw further diversification: *Leibian Zhu Shi Jiyan Yifang (Zhu’s Classified Empirical Medical Formulas)* mandated seven cycles of saltwater-soaking (“*yan shui qi pao fa*”, 盐水七泡法), followed by umbilicus removal, while the *Sheng Ji Zong Lu* (*Imperial Grace Formulary*) standardized thin slicing and sun-drying (“*bo qie pu gan*”, 薄切曝干). Adjuvants broadened to include ginger juice (*shengjiang zhi*, 生姜汁) and alum-water (*fan shui*, 矾水, mentioned in *Xiao’er Weisheng Zongwei Lunfang, General Treatise on Pediatric Health*). Jin–Yuan innovations added soapberry water (*zaojia shui*, 皂荚水) soaking (*Danxi Xin Fa* - *Danxi’s Essentials*) and rice-washed water (*mi gan shui*, 米泔水) soaking (*Chuangyang Jingyan Quanshu,* Complete Book on Sore Experiences).

Ming-Qing texts systematized these practices. The *Bencao Gangmu* (*Compendium of Materia Medica*) categorized processing with boys’ urine, black soybeans, and salt as the primary methods. The *Michuan Yinzhi Bencao Dacheng Yaoxing Fu* (*Secret Rhyme-Annotated Compendium* of Materia Medica Properties) quantified the ratios (e.g., 10 *liang* botanical drug: five *liang* black soybean: six *sheng* water). The *Xingyuan Sheng Chun* (*Apricot Garden’s Spring*) intensified the saltwater method to seven cycles with umbilicus removal.

Modernization emerged in the Republic of China era, with the *Danfang Jinghua* refining dynamic urine-soaking involving daily scrubbing. Today, the Chinese Pharmacopoeia (2020 Edition) retains methods involving boiling water rinsing, alum-water soaking, and ginger juice treatment, preserving principles established over millennia—utilizing flowing water, cycling adjuvants, and aiming for quantitative precision to balance the efficacy and safety.

#### 4.1.3 Fire-based processing (*Huo Jiagong*)

Fire-based processing (*Huo Jiagong*, 火加工) of Fuzi (附子) involves direct or indirect heating, driven by the dual goals of reducing the toxicity and preserving the therapeutic efficacy.

Northern and Southern Dynasties (420 CE–589 CE): The *Leigong Paozhi Lun (Master Lei’s Treatise on the Processing of botanical drug)* systematized Fuzi preparation by emphasizing morphological refinement and precision cutting. The key steps included scraping off attached buds/protrusions (“*gua shang yun zi*”, 刮上孕子) and removing base tips (“*qu di jian*”, 去底尖) to standardize the shape and thin slicing (“*bo qie*”, 薄切) to control the slice thickness. These practices reflected a sophisticated understanding of how morphology influences efficacy and further processing.

Song Dynasty (960 CE–1279 CE):

Northern Song: The *Michuan Yanke Longmu Lun* (*Secret Teachings on Ophthalmology* by Dragon Tree) established differentiated processing principles based on the intended use: raw use required peeling and tip removal (“*sheng yong qu pi jian*”, 生用去皮尖), while processed use involved fire-cracking (“*shu yong huo pao lie*”, 熟用火炮裂) for detoxification. This represented the first standard differentiating raw vs. processed Fuzi based on clinical application needs.

Southern Song: The *Shi Bian Liang Fang* (*Ten Convenient Formulas*) refined a four-step protocol: fire-cracking (“*pao lie*”, 炮裂) → umbilicus removal (“*qu pi qi*”, 去皮脐) → filing (“*cuo*”, 剉) → baking (“*bei*”, 焙), enhancing procedural systematization.

Ming and Qing Dynasties (1368 CE–1912 CE): The *Yao Jian* (Mirror of Medicinals) from the Qing Dynasty introduced quantitative parameters, specifying peeling aconite to a standardized weight/thickness equivalent to “one and a half *qian*” (“*qu pi qian ban*”, 去皮钱半), thus transforming empirical experience into measurable criteria.

Republic of China Era (1912–1949 CE): The *Danfang Jinghua* (Essence of Elixir Formulas) pioneered biological fermentation by soaking aconite in boys’ urine for 3 days and nights (“*tong bian jin san zhou ye*”, 童便浸三昼夜). This method likely leveraged the enzymatic activity to enhance the extraction or transformation of toxic metabolites, exemplifying an early integration of traditional techniques with biological principles.

#### 4.1.4 Fire–water co-processing *(Huo Shui Jiagong)*

Fire-water co-processing (*Huo Shui Jiagong*, 火水加工) combines heat with water or liquid adjuvants, encompassing techniques such as steaming, decoction, liquid stir-frying, and liquid-infusion methods (*Fu Zhi Fa*). For Fuzi (附子), decoction, often enhanced with adjuvants, is the most common method. Early precedents exist from the Northern and Southern Dynasties. *Leigong Paozhi Lun* described using specific fire (willow wood ash) followed by soaking in stream water with black soybeans during the “yin preparation” (*Yin Zhi*) phase, indicating combined thermal and liquid treatments.

The Southern Song Dynasty witnessed more defined methods. *Xiao’er Douzhen Fanglun* (Treatise on Smallpox and Rash Formulas for Children) pioneered a three-stage process: baking → hot urine soaking → ash roasting. *Sanyin Ji Yibing Zheng Fanglun* (Treatise on Triple Causes Unified Illness Patterns) introduced “seven cycles of saltwater soaking and roasting” (“*yan shui qi jin qi pao*”, 盐水七浸七炮”), iteratively processing Fuzi with salt broth and heat. The Yuan Dynasty’s *Yong Yao Xin Fa* advocated pretreatment with wine-washing and sun-drying.

Ming Dynasty techniques became more structured. *Bencao Zheng* (Rectifying the Materia Medica) detailed prolonged soaking in concentrated licorice (*Glycyrrhiza uralensis* Fisch. ex DC., Fabaceae) decoction followed by gentle frying until a “slightly spicy taste when chewed” (*kou jiao wei la*) remained. Other methods included boiling in boys’ urine (*Bencao Danfang*) and simmering with ginger juice and salt broth (*Chishui Xuanzhu,* Pearl of the Red River), emphasizing “synergistic adjuvant interactions” (*Fu Liao Xiang Shi*).

Qing Dynasty methods further refined these processes. *Bencao Gangmu Shiyi* (Supplements to the *Compendium of Materia Medica*) recorded “nine cycles of steaming and sun-drying with boys’ urine” (*Tongbian Jiu Zheng Jiu Shai*) for iterative detoxification. *Qi Shi Yi’an* (Qi’s Medical Case Studies) established the material selection criteria (“lotus-petal aconite”) and introduced “white star testing” (*Bai Xing Jian Ce*) as a quality check after roasting. Techniques such as dough-wrapped roasting (*Shoushi Baoyuan*) and ginger juice-steaming (*Mujing Dacheng*) adopted “no numbness upon tongue tasting” (*She Chang Bu Ma*) as the ultimate toxicity endpoint, embodying the philosophy of “harmonizing fire and water to counteract toxicity with nature” (*Shui Huo Xiang Ji, Yi Xing Zhi Du*).

Critical assessment of historical processing evidence: While the historical record documents a rich diversity of processing methods developed empirically over nearly two millennia, several critical limitations must be acknowledged. First, the vast majority of classical protocols lack standardized toxicity endpoints; subjective sensory markers such as “no tongue numbness” (*she chang bu ma*) or “white star disappearance” (*bai xing*) served as the primary quality-control indicators, which cannot be directly correlated with modern pharmacological safety parameters such as LD_50_ or electrocardiographic arrhythmia thresholds. Second, no classical source provides quantitative measurements of the DDA or MDA content before and after processing, making it impossible to evaluate the detoxification efficiency by modern standards. Third, the near-complete absence of documented processing failures or adverse outcomes in classical texts introduces a survivorship bias that may overestimate the historical processing efficacy. Finally, many adjuvant-based methods (e.g., boys’ urine soaking and soapberry water) have never been subjected to controlled experimental validation. Recent comprehensive reviews have similarly noted the disconnect between traditional processing knowledge and modern evidence-based toxicological evaluation (He et al., 2023; Wang. et al., 2023). Systematic experimental validation of historically prominent methods—particularly urine soaking, salt-water cycling, and licorice-assisted decoction—using modern analytical techniques (e.g., UPLC-MS/MS and DESI-MSI) and standardized animal models remains an important priority for future research (Liu. et al., 2022).

### 4.2 Advances in the current processing methods

#### 4.2.1 Potential processing mechanisms

**FIGURE 4:**
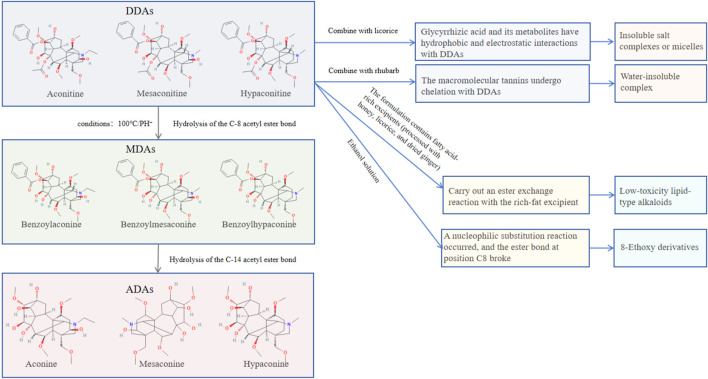
Integrated conceptual diagram of the major detoxification mechanisms during Fuzi processing.

Fuzi (*Aconitum carmichaelii* Debeaux, Ranunculaceae) exhibits broad pharmacological effects, including cardiotonic, antioxidant, antitumor, arterial smooth muscle modulation, endothelial apoptosis inhibition, suppression of cancer cell proliferation and metastasis, and analgesic properties. Its primary toxic metabolites are DDAs, which also account for its severe toxicity, thereby limiting clinical application—a characteristic dual nature of efficacy and toxicity. The primary target organ of DDA toxicity is the heart, with clinical manifestations encompassing limb numbness, consciousness impairment, arrhythmias, and potentially fatal heart failure in severe cases (Hu et al., 2024). Through historical refinement of processing methods, modern research has identified key detoxification mechanisms: hydrolysis, metabolite loss, chemical conjugation, reaction environment modulation, and ester-bond nucleophilic substitution (Figure 4).

##### 4.2.1.1 Hydrolysis

DDAs, the most toxic and pharmacologically potent metabolites in Fuzi, undergo detoxification via hydrolysis during processing. This converts DDAs into monoester alkaloids and hydrophilic aminoalcohol alkaloids. DDAs undergo hydrolysis and hydroxyl-catalyzed reactions under alkaline conditions (C8 deacetylation → C14 debenzoylation), ultimately yielding benzoylaconine (BA, a monoester alkaloid) and aconine (an aminoalcohol alkaloid) (Bao, et al., 2009). Hydrolysis rates are negatively correlated with ionic strength. In acidic aqueous solutions at 95 °C, hydrolysis remained limited despite accelerated partial degradation, with DDAs remaining predominant. Neutral to weakly acidic conditions produced minimal BA without complete hydrolysis. Temperature critically influences DDA hydrolysis; Gong, et al. (2017) reported significantly lower hydrolysis rates of DDAs in aqueous solutions at 50 °C. After 30 min, degradation rates of MA, HA, and AC reached 18.72%, 4.85%, and 28.91%, respectively, which increased to 33.3%, 6.99%, and 40.14%, respectively, at 1 h. At 100 °C, hydrolysis accelerated markedly, achieving complete degradation of all three DDAs within 30 min. Notably, recent studies have revealed that AC hydrolysis proceeds through three independent pathways—yielding indaconitine, benzoylaconitine (and subsequently aconine), and pyroaconitine—and that the hydrolysates of the different pathways cannot interconvert, with pyroaconitine having the least toxicity (He et al., 2022). This finding refines the classical two-step hydrolysis model (C8 deacetylation → C14 debenzoylation) and has important implications for optimizing the processing conditions to favor less toxic hydrolysis products.

##### 4.2.1.2 Metabolite loss

Another critical detoxification mechanism involves metabolite loss, which is achieved through peeling, coring, or prolonged soaking to eliminate alkaloids or facilitate their dissolution, thereby maintaining alkaloid content within safe thresholds. Toxic metabolites such as AC in Fuzi are predominantly concentrated in the epidermis and pith, and peeling and coring effectively remove most of the toxic substances. Research has demonstrated that peeling raw Fuzi slices reduces the content of three DDAs by approximately 50% or more (Hu, 2020). Further studies indicate that processing Fuzi with dried ginger (*Zingiber officinale* Roscoe, Zingiberaceae) enhances the solubilization of aconite alkaloids, which is likely due to the low water content of dried ginger, high boiling points of its volatile oils, and the synergistic action of gingerols (Ye et al., 2013). However, it has also been cautioned that co-decoction of Fuzi and dried ginger reduces the total aconite alkaloid content to approximately 36%, indicating that excessive alkaloid loss may compromise therapeutic efficacy (Chen, 2006).

##### 4.2.1.3 Chemical conjugation

Pharmacological studies confirm that the detoxification properties of licorice (*Glycyrrhiza uralensis* Fisch. ex DC., Fabaceae) primarily stem from the presence of triterpenoid derivatives (e.g., glycyrrhizic acid) and flavonoid polyphenols. Glycyrrhizic acid undergoes hydrolysis *in vivo* and *in vitro* to yield glycyrrhetinic acid and two glucuronic acid moieties. Research has demonstrated that increasing the proportion of licorice in Fuzi–licorice decoctions significantly reduces the residual diester alkaloid levels (e.g., AC) (Gao et al., 2020). This detoxification mechanism is caused by hydrophobic and electrostatic interactions between glycyrrhizic acid (and its metabolites) and DDAs, forming poorly soluble salt complexes that reduce the bioavailability of toxic metabolites. Further investigations have identified that glycyrrhizic acid interacts with DDAs to form micelles, which modulate hydrolysis kinetics through sustained release, enhanced solubility, and altered membrane permeability (Zhao X. et al., 2024). Additionally, studies have revealed that co-processing Fuzi with rhubarb (*Rheum palmatum* L., Polygonaceae) induces chelation between macromolecular tannins in rhubarb and DDAs, generating water-insoluble complexes that reduce the diester alkaloid content and mitigate toxicity (Yue, et al., 2007). Collectively, these conjugation mechanisms—micelle formation with glycyrrhizic acid, chelation with tannins, and ion-pair formation—represent distinct but complementary strategies through which traditional adjuvants reduce the bioavailability of toxic DDAs.

##### 4.2.1.4 Biotransformation

The core principle of Fuzi biotransformation technology involves utilizing selected microbial strains to act on pretreated Fuzi substrates (e.g., powder or extracts). These microorganisms employ extracellular enzymes or intracellular biocatalytic systems generated during metabolism to selectively modify the structure of aconite alkaloids. Studies have indicated that this process effectively reduces the concentration of toxic DDAs by 60%–80%, while specifically hydrolyzing the C8 ester bond to generate pharmacologically active intermediates such as MDAs (Jiang, et al., 2012).

##### 4.2.1.5 Ester-bond nucleophilic substitution

DDAs in Fuzi exhibit structural instability under specific physicochemical conditions, with the primary degradation pathways including thermal decomposition, hydrolysis, and ester exchange reactions with hydroxyl-containing reagents. Yue et al. discovered that when Fuzi is combined with honey-fried licorice, white peony root (*Paeonia lactiflora* Pall., Paeoniaceae), or dried ginger—botanical drugs rich in fatty acids—ester exchange reactions readily occur (Yue et al., 2007). These reactions convert highly toxic diester alkaloids into less toxic lipid-type alkaloids, thereby achieving detoxification. However, the precise reaction mechanisms remain unclear and require further investigation.

Experimental studies have demonstrated that MA, AC, and HA undergo cleavage of the C8 ester bond in ethanol solutions, generating corresponding 8-ethoxy derivatives via nucleophilic substitution (Zhang and Zhu, 2016). This reaction mechanism not only elucidates the structural regulatory effect of the ethanol solvent on DDAs but also provides directional guidance for developing detoxification processes in Fuzi—specifically, targeting the cleavage of highly toxic ester bonds to achieve toxicity reduction.

#### 4.2.2 Processing techniques

As a toxic botanical drug, the processing of Fuzi (*Aconitum carmichaelii* Debeaux) must adhere to the principle of “toxicity reduction while preserving efficacy,” which is critical for ensuring clinical safety. The theoretical framework for Fuzi processing was established in the *Jingui Yuhan Jing* (*Golden Chamber and Jade Casket Classic*), which proposed “seven detoxification methods through processing.” Modern techniques have diversified traditional practices, and this section summarizes both the conventional and contemporary methods along with their associated detoxification mechanisms.

##### 4.2.2.1 Heishunpian (black sliced aconite) processing

The preparation of HSP integrates two primary detoxification mechanisms: hydrolysis of toxic DDAs and metabolite loss through leaching. However, traditional methods lack standardized quantification for critical parameters such as bitter brine (*danba ye*) concentration, rinsing–boiling cycles, and steaming duration. Recent advancements in multicriteria optimization have addressed these limitations. Gong et al. (2022) employed the analytic hierarchy process-entropy weight (AHP-EW) method to standardize the processing parameters, while Wang et al. (2021) utilized membership degree analysis to identify an optimized protocol involving 7-day bitter brine immersion, three rinsing cycles, and 150-min steam treatment. Research has further demonstrated that extending the duration of brine immersion beyond 20 days significantly reduces DDA content (e.g., AC, MA and HA) (Hou, et al., 2020). Notably, the trade-off between detoxification and bioactive metabolite loss during brine treatment remains controversial. Bitter brine, which is primarily composed of calcium chloride, sodium chloride, potassium chloride, and magnesium chloride, exhibits potential neurotoxic effects. Consequently, modern research emphasizes the need for further exploration of brine concentration and immersion duration to balance safety and therapeutic efficacy.

##### 4.2.2.2 Baifupian (BFP) processing

Research by Ye et al. revealed significant regional variations in the total alkaloid content of BFP (Ye et al., 2019). Specifically, BFP from the Yunnan region contained 0.244%–0.440% total alkaloids, with MDAs accounting for 0.0326%–0.1215% and DDAs accounting for 0.0071%–0.0199%. In contrast, BFP from the Jiangyou region contained 0.070%–0.230% total alkaloids, comprising 0.0101%–0.0158% MDAs and only 0.0008%–0.0034% DDAs. BFP from the Shaanxi region showed 0.128% total alkaloids, with 0.0230% MDAs and 0.0153% DDAs. Building on this, the study compared the processing effectiveness of alum-free versus traditional alum-processed BFP. The alum-free BFP exhibited higher total alkaloid and MDA contents compared to the traditional alum-processed BFP, which might be attributed to the lack of extensive rinsing/leaching in the alum-free method. However, its detoxification capacity (i.e., reduction of DDAs) was relatively weaker. Therefore, it is inferred that BFP processing technology involves three primary detoxification mechanisms: metabolite loss (leaching), hydrolysis, and peeling.

##### 4.2.2.3 Yanfuzi (salt-processed Fuzi) processing

The current practice of soaking Fuzi in salt brine (*Danba*) during processing has raised concerns due to potential adverse effects, including gastrointestinal irritation and inhibition of the cardiovascular and nervous systems. Consequently, Zhao et al. analyzed four alternative salt solutions as substitutes for *Danba* in salt-soaking experiments (Zhao Y.-H et al., 2024). They observed that at low salt concentrations, Fuzi quality was unstable, with decomposition occurring within 5–10 days. However, upon extending the soaking period, an average reduction of approximately 70% in the DDA content was detected at 21 days. Furthermore, when fresh Fuzi was soaked separately in CaCl_2_, MgCl_2_, or NaCl solutions, the loss of DDAs was relatively less compared to that with the traditional *Danba* soaking treatment (Zhang K. et al., 2020). This indicates that using specific single-salt solutions might prevent the excessive loss of DDA metabolites that is often associated with salt brine soaking.

##### 4.2.2.4 Danfupian (desalted/light-colored aconite slices) processing

Danfupian is obtained by thoroughly rinsing Yanfuzi, (salt-processed Fuzi) to remove the salt, followed by co-processing with black soybean (Heidou; *Glycine max* (L.) Merr., Fabaceae) and licorice (Gancao; *Glycyrrhiza uralensis* Fisch. ex DC., Fabaceae). Its detoxification mechanism is potentially related to the chemical conjugation initiated by co-processing with black soybean and licorice. A study analyzed the characteristic chromatogram of Danfupian and compared it with those of Fuzi processed solely with black soybean, licorice, or plain water (Guo et al., 2015). The results indicated that, compared to Yanfuzi, one metabolite was absent in Danfupian, which was likely lost during the rinsing process. Conversely, eight new metabolites were present, three of which were identified as originating from the adjuvants (black soybean and licorice). This indicates that the significant reduction in Danfupian’s toxicity can be attributed to the influence of these adjuvants on its material composition and metabolite profile.

##### 4.2.2.5 High-pressure steaming processing

High-pressure steaming utilizes elevated pressure and temperature to promote the hydrolysis of toxic metabolites in Fuzi, thereby effectively reducing the toxicant levels while minimizing the loss of potentially active metabolites. Zhang C. et al. (2020) employed a steaming process at 0.245 MPa for 1.5 h. When administered to mice at doses of hundreds of times the clinical adult dosage, no related toxicity was observed. Research utilizing an orthogonal design-based process optimization model revealed that steaming at 120 °C for 12 h significantly decreased the content of DDAs (0.04 mg/g–0.08 mg/g) in Fuzi, while the content of MDAs (0.70 mg/g–0.90 mg/g) increased slightly, and the total alkaloid content reduced slightly (Lin et al., 2014). These findings are consistent with those of recent studies confirming that pressurized steaming at approximately 120 °C achieves a DDA detoxification rate exceeding 90% while preserving the cardiotonic activity of MDAs (Li et al., 2025), supporting the potential of high-pressure steaming as a standardized modern alternative to traditional processing.

##### 4.2.2.6 Microwave processing

Microwave processing of Fuzi involves slicing washed raw Fuzi roots into thin pieces (approximately 3 mm), sealing them with water (enough to cover the slices), setting the temperature to 90 °C, heating with microwave for 90 min, and then continuing with microwave vacuum frequency conversion drying at 60 °C until dry. This processing method utilizes the high-frequency vibration of water molecules and their intense collisions with surrounding molecules to generate substantial heat, inducing the hydrolysis of DDAs within the Fuzi slices and thereby achieving safe detoxification (He G. et al., 2023). Concurrently, during the drying process, the synchronous internal and external heating characteristics of microwaves causes a rapid increase in temperature inside the slices, thus generating significant steam, forming pressure gradients, and extruding outward. This causes the formation of numerous micropores within the slices, leading to a porous structure and loose texture. Research has analyzed the factors influencing microwave processing of Fuzi. Slice thickness, water content, processing time, and microwave power level all affect the processing outcome and alkaloid content (He G. et al., 2023). Using total alkaloid change as an indicator, optimal conditions for thorough detoxification included raw Fuzi slice thickness of 3 mm, rehydration rate of 60%–80%, and processing time adjusted inversely with increasing microwave power. Jia et al. (2016) discovered significant transformations upon analyzing the alkaloid content in microwave-processed Fuzi slices: DDAs such as MA content decreased from 1.1435 mg·g^-1^ to 0.0797 mg·g^-1^, that of HA decreased from 0.03361 mg·g^-1^ to 0.05927 mg·g^-1^, and AC (original concentration 0.1910 mg·g^-1^) became undetectable after processing. Conversely, content of MDAs such as benzoylmesaconitine (BMA) increased from 0.6558 mg·g^-1^ to 0.7329 mg·g^-1^, that of benzoylhypaconitine (BHA) increased from 0.0700 mg·g^-1^ to 0.0837 mg·g^-1^, and that of benzoylaconitine (BAC) increased from 0.0257 mg·g^-1^ to 0.0905 mg·g^-1^.

##### 4.2.2.7 Microbial fermentation processing

The active substances produced by the microbial strain can selectively decompose toxic metabolites while promoting the generation of less toxic but pharmacologically active metabolites, reducing the toxicity–efficacy ratio by 83% (Li et al., 2019)*. Paecilomyces variotii* Bainier (宛氏拟青霉) was tested through research on the targeted biotransformation of Fuzi. This strain demonstrated significant efficacy that overcomes challenges inherent in traditional processing, achieving the dual effects of detoxification and efficacy in degrading toxic aconite alkaloids. Fermentation experiments demonstrated that after 48 hours of cultivation under optimized conditions, this strain significantly reduced toxic diester diterpenoid alkaloid (DDA) content and increased lower-toxicity monoester diterpenoid alkaloid (MDA) proportion, thus providing an innovative approach for TCM processing.

Recent advances and clinical safety considerations: Since 2022, several comprehensive reviews and experimental studies have enriched the understanding of Fuzi processing and its clinical implications. Liu et al. (2022) applied DESI-MSI combined with metabolomics to visualize the spatiotemporal dynamics of DDA-to-MDA conversion during steaming, identifying 4.0 h as the optimal processing duration and 42 metabolic markers distinguishing raw from processed Fuzi. He Y. X. et al. (2023) and Wang et al. (2023) independently reviewed the chemical metabolites, processing methods, and compatibility strategies of Fuzi, emphasizing that proper processing and appropriate botanical drug compatibility can reduce the DDA content by over 90%. More recently, Liang et al. (2025) provided a comprehensive overview highlighting that despite decades of research, the precise molecular mechanisms of adjuvant-mediated detoxification remain incompletely understood. Li et al. (2025) introduced the concept of “integrated toxicology” for *Aconitum*, advocating a shift from qualitative safety assessment toward quantifiable and controllable toxicity evaluation. Xiang et al. (2025) comparatively analyzed three pharmacopoeial Fuzi products using UPLC-MS, demonstrating significant interproduct differences in the metabolite profiles and confirming that processing substantially reduces the DDA content while increasing MDA and polysaccharide levels. From a clinical safety perspective, ongoing post-marketing surveillance continues to report sporadic cases of AC-related arrhythmia associated with insufficiently processed or improperly prescribed Fuzi-containing formulations, underscoring the persistent gap between standardized pharmacopoeial processing and real-world clinical practice. Compared with these recent reviews, the present work uniquely contributes a diachronic analysis that systematically correlates the historical evolution of processing methods with the parallel development of toxicity understanding across Chinese dynasties, thereby providing an ethnopharmacological framework that contextualizes modern processing science within its historical origins.

## 5 Discussion, limitations, and future perspectives

### 5.1 Critical assessment of the reviewed evidence

#### 5.1.1 Limitations and future research needs

Several cross-cutting methodological limitations characterize the reviewed body of literature. First, most studies have used acutely toxic or supra-therapeutic dose levels, and there is a near-complete absence of chronic exposure studies (>90 days); this is particularly problematic given that Fuzi is prescribed in long-term TCM formulas. Second, the chemical characterization of Fuzi preparations varies considerably across studies: many publications, especially those predating 2020, do not quantify DDA and MDA contents, do not report the drug-to-extract ratio or extraction solvent, and do not provide batch information, all of which are required for reproducibility according to the ConPhyMP guidelines. Third, a substantial proportion of *in vitro* studies employed pure AC at concentrations (50 μM–200 μM) that substantially exceed clinically achievable plasma levels, and several others used poorly characterized crude extracts without chemical profiling, limiting the interpretability of both types of data. Fourth, two studies employing network toxicology or molecular docking approaches (Zhang K. et al., 2020; Yin et al., 2024) lack sufficient experimental validation; in accordance with the Four Pillars requirement, their mechanistic conclusions should be considered hypothesis-generating rather than confirmatory. Fifth, all clinical toxicity data originate from retrospective poisoning case series, without systematic prospective pharmacovigilance data from controlled clinical use of processed Fuzi products. Finally, many studies reported only vehicle controls without positive controls, thus reducing confidence in the observed effect magnitudes.

#### 5.1.2 Limitations of processing studies

The evidence on Fuzi processing and detoxification, while extensive in scope, is characterized by several cross-cutting limitations. First, historical Materia Medica descriptions of the processing methods lack quantitative parameters (e.g., temperature, duration, and adjuvant-to-drug ratios), standardized toxicity endpoints, and reproducibility criteria, making it difficult to evaluate their detoxification efficacy by modern standards. Second, among modern processing studies, the chemical characterization of starting materials and processed products varies substantially: many investigations do not report the baseline DDA/MDA content of raw Fuzi, extraction solvent, or drug-to-extract ratio, preventing meaningful cross-study comparison. Third, the Chinese Pharmacopoeia (2020 edition) stipulates product-specific DDA ceiling values for processed Fuzi preparations (e.g., ≤0.020% for HSP and BFP), along with a minimum MDA content requirement of ≥0.010%, but this does not address the retention or loss of potentially therapeutic aminoalcohol alkaloids, nor does it fully account for batch-to-batch variability in raw material composition arising from geographical origin and processing heterogeneity. Fourth, no head-to-head comparative studies evaluating the full-spectrum toxicological profiles (beyond DDA content) of different processing methods (e.g., high-pressure steaming vs. traditional salt-processing vs. microbial fermentation) have been conducted under standardized conditions. Finally, virtually all processing efficacy data are based on chemical analysis (DDA/MDA quantification) or acute toxicity testing (LD_50_), with a conspicuous absence of subacute or chronic safety data for processed products, and there are no prospective clinical pharmacovigilance studies comparing the safety of different processed Fuzi preparations in patients.

### 5.2 Contemporary clinical safety challenges of Fuzi-containing formulations

Although traditional processing methods substantially reduce the acute lethality of Fuzi by converting highly toxic DDAs into MDAs and ADAs (see Section 3), several contemporary clinical safety challenges remain unresolved and warrant critical discussion.

#### 5.2.1 Highly individualized cardiotoxicity

Emerging evidence indicates that AC-induced cardiotoxicity exhibits pronounced interindividual variability that cannot be predicted from the dose alone. Using diversity outbred (DO) mice—a genetically heterogeneous model designed to recapitulate human population-level variation—Guo et al. demonstrated that cardiac injury markers showed variation coefficients of 34.08%–53.17% across animals receiving the same oral AC dose (0.3 mg·kg^-1^·d^-1^–0.9 mg·kg^-1^·d^-1^ for 7 days) and identified hemoglobin subunit beta (HBB) as a novel susceptibility biomarker mediating differential cardiotoxicity via the ABHD5/AMPK/HDAC4 axis (Guo, et al., 2024). This finding has direct clinical implications; patients with certain hemoglobin variants or hemolytic conditions may face elevated risk even when receiving correctly processed Fuzi products at pharmacopoeial doses. However, the DO mouse model, while genetically diverse, does not fully replicate the complexity of human pharmacogenomics, and the HBB–cardiotoxicity association has not been confirmed in clinical cohorts. Prospective genotype-stratified pharmacovigilance studies are, therefore, needed before HBB can be adopted as a clinical risk biomarker.

#### 5.2.2 Persistent poisoning risk from inadequately processed or misused preparations

Despite pharmacopoeial quality standards, AC poisoning remains a recurrent public health problem. A forensic toxicological analysis of 25 AC-induced deaths received by the Academy of Forensic Science (China) from 2005 to 2023 found that the majority of the fatalities resulted from misuse of the botanical drug—particularly the consumption of homemade medicinal liquors containing unprocessed or improperly processed *Aconitum* material—with postmortem blood AC levels ranging from 2.9 to 470 ng/mL (Wang, et al., 2025). A 2025 clinical case series reported eight patients who accidentally ingested AC-containing Chinese patent medicines, all of whom presented with GI symptoms followed by cardiac arrhythmias; one patient experienced cardiac arrest, requiring cardiopulmonary resuscitation and ECMO support, and all eight ultimately survived with appropriate treatment (Shang et al., 2025). A narrative review of aconite poisoning published in 2024 further emphasized that clinical manifestations encompass gastrointestinal, neurological, and life-threatening cardiac dysrhythmias that may not respond to standard antiarrhythmic therapies and that there is currently no specific antidote (Lawson et al., 2025).

Critically, these poisoning events share a common theme: they arise almost exclusively from non-medical misuse (homemade liquors, topical preparations taken orally, and raw material ingestion) rather than from correctly processed and prescribed Fuzi products used within pharmacopoeial guidance. This distinction is essential for an accurate risk assessment: the clinical safety of pharmacopoeia-compliant processed Fuzi (e.g., HSP and Paofupian) should not be conflated with the extreme toxicity of raw or improperly handled aconite material. Nevertheless, the continued occurrence of such events underscores the need for stronger regulatory enforcement, patient education, clear labeling, and point-of-dispensing safeguards.

#### 5.2.3 The standardization dilemma: the efficacy–toxicity trade-off in processing

A fundamental clinical dilemma in Fuzi processing is that over-processing eliminates not only toxic DDAs but also pharmacologically active MDAs and other bioactive metabolites. Sun et al. demonstrated that even the 120-min decoction (FZ-120), while abolishing acute lethality in mice, still induced subclinical hepatotoxicity detectable only by histopathology and that UPLC-MS revealed residual AC-type alkaloids below the detection limit of conventional HPLC (Sun et al., 2018). More recent metabolomic profiling of different processed Fuzi varieties (HSP, BFP, and Yanfuzi) has confirmed marked product-to-product variation in the DDA/MDA/AA ratio, indicating that the “safety” and “efficacy” of a given processed product depend critically on the specific processing protocol, batch of raw material, and analytical sensitivity of the quality-control method employed (Liang et al., 2025). The Chinese Pharmacopoeia (2020 edition) mandates a total DDA limit of ≤0.020% and a minimum total MDA content of ≥0.010% for processed Fuzi products, but it does not set a minimum specification for retained MDAs of individual alkaloid types—meaning that an overprocessed product may pass quality testing yet offer diminished therapeutic benefit for conditions such as chronic heart failure.

#### 5.2.4 Absence of prospective clinical safety data from therapeutic use

Perhaps the most critical gap in the evidence base is the near-complete absence of prospective, controlled clinical safety data for processed Fuzi products used at therapeutic doses. All available clinical toxicity information is derived from retrospective poisoning case series or *post hoc* analyses of adverse events in clinical trials where Fuzi-containing formulas (e.g., Shenfu injection) were co-administered with conventional medicines. For instance, a 2022 systematic review and meta-analysis of Shenfu injection (SFI) in post-acute myocardial infarction heart failure cases pooled data from multiple RCTs and reported a lower adverse-event rate in the SFI group (9.73%) compared with conventional therapy alone (21.7%), with common reactions limited to facial flushing, dry mouth, and mild nausea (Wu et al., 2022). A more recent meta-analysis of SFI for bradyarrhythmia in 2025 similarly found the adverse event incidence to be 5.25% in the SFI group versus 34.04% in the control group, with no serious adverse events attributed to SFI (Wei et al., 2025). While these data are reassuring, critical appraisal reveals that (a) none of the included RCTs were specifically designed to evaluate SFI safety as a primary endpoint, (b) most were single-center studies with small sample sizes, and (c) causal determination of adverse reactions in combination-therapy settings was not performed. Consequently, the overall quality of evidence for SFI safety was rated “very low” to “moderate” according to the GRADE assessment (Wu et al., 2022).

No prospective pharmacovigilance study has systematically monitored patients receiving oral processed Fuzi products (decoction slices) at therapeutic doses over extended periods (>90 days). Given that Fuzi is prescribed in long-term TCM formulas for chronic conditions such as heart failure, rheumatoid arthritis, and chronic kidney disease, the absence of chronic-use safety data—including monitoring for cumulative hepatotoxicity, nephrotoxicity, and reproductive effects—represents a significant evidence gap that urgently requires attention.

### 5.3 Comparison with existing reviews and the novelty of this work

Multiple reviews on *Aconitum carmichaelii* and Fuzi have been published since 2022, each addressing different aspects of its chemistry, pharmacology, toxicology, or processing. To contextualize the scope and contribution of the present work, a systematic comparison with the most relevant recent reviews is presented below (Table 4).

**TABLE 4:** Systematic comparison of the present review with recent Fuzi/*Aconitum carmichaelii* reviews (2022–2025).

Dimension	Fu, et al. (2022)	Wang, et al. (2023)	He Y. X. et al. (2023)	Jiang, et al. (2022)	Wu, et al. (2024)	Liang, et al. (2025)	This review
Primary focus	Polysaccharides and phenolic compounds in Fuzi roots	Ethnopharmacological use, pharmacology, toxicology, and processing	Chemical metabolites, pharmacology, toxicology, processing, and compatibility	Aconitine cardiotoxicity mechanisms (meta-analysis)	Fuzi for kidney disease: phytochemistry, processing, and pharmacology	Chemical metabolites, pharmacology, toxicity, detoxification, and applications	Integrated historical–modern processing and full-spectrum toxicology
Diachronic historical analysis	None	Brief historical context	Brief introductory mention	None	None	Brief overview	Comprehensive: Han Dynasty to Qing Dynasty with primary-source analysis
Toxicology coverage	Minimal (polysaccharide focus)	Cardiotoxicity and neurotoxicity emphasized; toxicity in other organs briefly noted	Cardiotoxicity, neurotoxicity, reproductive toxicity, hepatotoxicity, and embryotoxicity listed	Cardiotoxicity only (13 preclinical studies)	Cardiac, hepatic, renal, and neuro (focus on kidney disease)	General toxicity overview	Full-spectrum: cardiac, hepatic, renal, neuro, developmental, reproductive, GI, genotoxicity, and subacute/chronic, each with critical appraisal
Processing mechanism analysis	Not covered	Processing effects on chemistry described	Processing and compatibility discussed	Not applicable	Processing techniques and phytochemical changes	Detoxification mechanisms described	Mechanistic integration: hydrolysis, conjugation, ester-bond substitution, and metabolite loss; with historical evolution and schematic pathway
Critical appraisal of primary studies	Limited	Absent	Absent	Systematic (PRISMA-compliant meta-analysis)	Limited	Limited	Explicitly applied: Four Pillars framework, ConPhyMP guidelines, study-level critique in each toxicity subsection
Clinical safety of processed products	Not addressed	Not addressed	Not addressed	Not addressed	Briefly mentioned	Briefly mentioned	Dedicated discussion with 2023–2025 clinical data
Stated search strategy and inclusion/exclusion criteria	Keywords listed, with no formal criteria	Databases listed, with no formal criteria	Not reported	PRISMA-compliant	Databases listed, with no formal criteria	Not reported	Full M&M: search strings, databases, inclusion/exclusion criteria, and quality assessment framework

#### 5.3.1 Specific limitations of the existing reviews

Fu et al. (2022) provided a valuable systematic account of polysaccharides and phenolic metabolites in *A. carmichaelii* roots but explicitly excluded alkaloid toxicology from its scope, leaving the most clinically urgent safety questions unaddressed. Wang et al. (2023) presented a broader overview encompassing ethnopharmacological use, pharmacology, toxicology, and processing but did not report a formal search strategy, inclusion/exclusion criteria, or quality assessment framework; its toxicology section focused primarily on cardiac and neural systems, with hepatotoxicity, nephrotoxicity, reproductive toxicity, and gastrointestinal toxicity receiving only cursory treatment. He Y. X. et al. (2023) similarly covered chemical metabolites, pharmacology, toxicology, processing, and compatibility but without the stated methodological rigor—its *Materials and Methods* section consisted of a single sentence listing databases, and no critical appraisal of primary study quality was performed. Jiang, et al. (2022) conducted the only PRISMA-compliant systematic review and meta-analysis in the field, but the study’s scope was restricted to preclinical evidence of AC-induced cardiotoxicity (13 studies) without addressing other organ toxicities, processing, or clinical data. Wu et al. (2024) provided a disease-oriented review focusing specifically on Fuzi for kidney disease, offering useful insights into salt-processing and pharmacokinetics but covering toxicology and processing only insofar as they relate to renal applications, thereby lacking the panoramic perspective required for a comprehensive safety assessment. Liang et al. (2025) delivered the most recent and broadest overview, summarizing chemical metabolites, pharmacological effects, toxicity, detoxification mechanisms, and applications, but they did not include a diachronic historical analysis of toxicity perception and processing evolution, did not explicitly apply critical appraisal frameworks (Four Pillars and ConPhyMP), and did not discuss contemporary clinical safety challenges of Fuzi-containing formulations in current medical practice.

#### 5.3.2 Novelty and distinctive contributions of this review

Against this landscape, the present review makes several distinctive contributions that address the identified gaps in the existing literature:

First, it constructs a comprehensive diachronic timeline integrating primary-source analysis of classical Chinese Materia Medica texts from the Han Dynasty through the Qing Dynasty, systematically tracing how empirical understanding of Fuzi’s toxicity and the corresponding detoxification strategies co-evolved over approximately two millennia. No existing review has performed this level of historical–ethnopharmacological analysis for *A. carmichaelii.*

Second, it provides the most comprehensive toxicological assessment to date, covering eight distinct organ-system or endpoint-specific toxicities—cardiotoxicity, hepatotoxicity, nephrotoxicity, neurotoxicity, developmental toxicity, reproductive toxicity and genotoxicity, gastrointestinal toxicity, and subacute/chronic toxicity—each supported by a structured summary (Table 2) and accompanied by study-level critical appraisal identifying specific methodological strengths and limitations.

Third, the *Materials and Methods* section (Section 2) provides full transparency regarding the search strategy, databases, search strings, inclusion and exclusion criteria, data extraction procedures, quality assessment framework (based on the Four Pillars of Best Practice for ethnopharmacology and the ConPhyMP guidelines for phytochemical characterization), and terminology conventions. This level of methodological transparency is intended to facilitate independent replication and critical appraisal and is consistent with the reporting standards advocated by the Four Pillars framework and the ConPhyMP guidelines.

Fourth, this review explicitly discusses contemporary clinical safety challenges of Fuzi-containing formulations, including highly individualized cardiotoxicity, the ongoing poisoning burden from misuse, the efficacy–toxicity trade-off inherent in processing, and the critical absence of prospective clinical pharmacovigilance data—a dimension that has been overlooked by all existing reviews.

Fifth, processing mechanisms are not merely cataloged but mechanistically integrated: the hydrolysis of DDAs (C8 deacetylation → C14 debenzoylation), chemical conjugation with adjuvant-derived metabolites (e.g., glycyrrhizin from *Glycyrrhiza uralensis* Fisch. ex DC., Fabaceae), and ester-bond nucleophilic substitution are synthesized into a unified detoxification framework across different traditional adjuvant-based processing methods (Table 5).

**TABLE 5:** Comparison of toxicity reduction across different Fuzi-processing methods.

Toxicity indicator	Raw Fuzi	Salt-processed Fuzi	Black soybean-processed Fuzi	Glycyrrhiza–ginger-processed Fuzi
LD_50_ (mouse, mg/kg)	0.8–1.2	15.6–20.3	18.5–23.7	22.4–28.9
DDA content (%)^1^	0.5–1.5 (aconitine, mesaconitine, etc.)	0.05–0.1	0.03–0.08	0.01–0.05
MDA content (%)^2^	<0.1	0.3–0.6	0.4–0.8	0.5–1.0
Toxicological mechanism	Strong Na^+^ channel blockade, inducing arrhythmia	Mild Na^+^ channel inhibition	Reduced Na^+^ channel affinity	Adjuvants synergistically inhibit toxic metabolism
Clinical manifestations	Arrhythmia, oral/lingual numbness, and shock	Occasional gastrointestinal discomfort	Mild numbness (rare)	High safety profile; adverse reaction rate <1%

In aggregate, by bridging deep historical–ethnopharmacological analysis with contemporary toxicological evidence, explicit methodological rigor, and clinical safety discussion, this review provides a resource that is both more comprehensive in scope and more critically grounded than any single existing review in the field.

### 5.4 Future research priorities

This paper proposes five prioritized research directions to advance the safe and evidence-based application of Fuzi in clinical practice.

Priority 1: Systematic experimental validation of under-explored historical processing methods. The historical survey (Table 3; Supplementary Table S1) reveals numerous processing variants that have never undergone modern analytical evaluation—including soapberry-water soaking (zaojia shui, Yuan Dynasty), rice-washed-water soaking (mi gan shui, Jin–Yuan period), and cinnabar-embedded roasting (Qing Dynasty). Systematic investigation of these methods using HPLC-MS/MS-based DDA/MDA/AA quantification, *in vitro* cytotoxicity assays (Nav1.5 channel inhibition and H9c2 cardiotoxicity models), and *in vivo* LD_50_ determinations could uncover detoxification mechanisms or adjuvant synergies that have been empirically exploited for centuries but remain scientifically uncharacterized. Findings from such validation studies would also provide the critical evidence needed to determine which historical methods merit standardization or clinical re-evaluation.

Priority 2: Multi-organ multi-endpoint toxicity assessment of processed Fuzi products. Current regulatory evaluation of processed Fuzi focuses almost exclusively on DDA ceiling values, while the comprehensive toxicological profile documented in Section 3.3 and Table 2—spanning cardiac, hepatic, renal, neural, and reproductive/developmental endpoints—remains incompletely addressed. Future studies should (a) conduct standardized 90-day repeated-dose toxicity studies of clinically relevant processed forms (HSP and BFP) in rodents, following ICH M3 (R2) guideline frameworks, to fill the near-complete absence of chronic toxicity data; (b) systematically characterize nephrotoxicity using modern renal injury biomarkers (KIM-1, NGAL, and cystatin C) and validated renal tubular cell lines (HK-2), which the literature has addressed only marginally; and (c) conduct female reproductive toxicity studies—including multigeneration designs—complemented by a standard regulatory genotoxicity battery (Ames test and *in vitro* chromosome aberration assay), given that the current data are confined almost entirely to male rodent sperm-damage endpoints.

Priority 3: Development of multi-parameter, toxicological-endpoint-informed quality standards. The Chinese Pharmacopoeia (2020 edition) stipulates a compositional threshold of total DDAs ≤0.020% and total MDAs ≥0.010% for processed Fuzi products, but it does not specify acceptable ranges for aminoalcohol alkaloids (ADAs), nor does it address batch-to-batch variability arising from the geographical origin or processing heterogeneity. Future standards should correlate specific DDA/MDA/AA ratio profiles with quantitative toxicological endpoints (e.g., LD_50_ in standardized rodent models and IC_50_ for Nav1.5 channel inhibition) across the full spectrum of organ toxicities identified in Section 3.3, moving beyond simple “toxic metabolite ceiling” approaches toward pharmacologically grounded, multiparameter quality control frameworks. All future studies should additionally report, at the minimum, the following harmonized chemical characterization data to enable cross-study comparison: individual DDA content (AC, MA, and HA), individual MDA content (benzoylaconitine [BAC], benzoylmesaconitine [BMA], and benzoylhypaconitine [BHA]), drug-to-extract ratio, extraction solvent, and batch information, in accordance with ConPhyMP guideline requirements.

Priority 4: Mechanistic elucidation of adjuvant-mediated and compatibility-based detoxification. Classical processing adjuvants, including *Glycyrrhiza uralensis* Fisch. ex DC. (Fabaceae), *Zingiber officinale* Roscoe (Zingiberaceae), black soybean (*Glycine max* (L.) Merr., Fabaceae), and salt brine, facilitate DDA transformation through diverse mechanisms: glycyrrhizic acid forms hydrophobic salt complexes with DDAs that reduce their bioavailability, macromolecular tannins (e.g., from *Rheum palmatum* L., Polygonaceae) generate water-insoluble chelates with DDAs, and ester-bond nucleophilic substitution by chloride ions in salt brine displaces the C8-acetyl group of DDAs. Despite these mechanistic insights, the precise kinetic parameters, structure–activity relationships, and *in vivo* pharmacokinetic consequences of these adjuvant–alkaloid interactions remain incompletely defined. Reaction kinetics modeling, stable-isotope labeling experiments, and molecular docking should be applied to delineate these interactions at the molecular level. Concurrently, the chemical and pharmacokinetic bases of classic Fuzi combinations documented in historical formulae [e.g., Fuzi–Ganjiang (dried ginger), Fuzi–Gancao (licorice), and Sini Tang] should be systematically re-examined to provide mechanistic justification—or evidence-based re-evaluation—of these traditional co-prescribing practices.

Priority 5: Prospective clinical pharmacovigilance and harmonized chemical biomonitoring. As critically appraised in Section 5.2, the existing clinical safety evidence base for Fuzi-containing formulations rests overwhelmingly on retrospective case reports and spontaneous adverse-event registries, without a single published prospective, controlled pharmacovigilance study. Establishing multicenter prospective observational cohorts with the following features represents the most important near-term translational research priority: (a) standardized serial plasma biomonitoring of AC, MA, HA, BAC, BMA, and BHA at clinically relevant time points; (b) pre-specified multi-organ safety endpoints including ECG (QTc), liver function tests (ALT and AST), renal markers (BUN, CRE, and KIM-1), and neurological assessments; (c) patient-level pharmacokinetic modeling incorporating CYP3A4/CYP2D6 genotyping and P-glycoprotein phenotyping to account for interindividual variability; and (d) harmonized processing quality documentation for all Fuzi preparations used, enabling preparation-type comparisons. In parallel, the development of validated pre-symptomatic biomarkers for AC-class cardiotoxicity—analogous in concept to cardiac troponin for myocardial injury—should be pursued as a foundational objective for future real-world safety surveillance.

### 5.5 Conclusion

This review demonstrates that the centuries-long empirical efforts to mitigate Fuzi toxicity through processing were, in essence, a pre-scientific optimization of DDA hydrolysis—a conclusion now supported by convergent modern phytochemical and toxicological evidence. By providing the first systematic historical timeline that directly correlates evolving toxicity perception with processing innovation, together with a comprehensive multisystem toxicological synthesis and a critical appraisal of contemporary clinical safety data, this work offers an integrative framework that can guide the development of pharmacologically grounded quality standards, inform the design of prospective pharmacovigilance studies, and serve as a methodological template for the evidence-based evaluation of other toxic botanical drugs in ethnopharmacological practice.

## Glossary

Fuzi: *Aconiti Lateralis Radix Praeparata*
TCM: Traditional Chinese medicine
DAs: Diterpenoid alkaloids
DDAs: Diester–diterpenoid alkaloids
MDAs: Monoester–diterpenoid alkaloids
ADAs: Aminoalcohol–diterpenoid alkaloids
MDA: Malondialdehyde
**LD**_**50**_: Median lethal dose
MTD: Maximum tolerated dose
MFD: Minimum fatal dose
HSP: Heishunpian (black sliced aconite)
BFP: Baifupian (white aconite slices)
NF: Nifupian
FZI: Fuzi injection
FAA: Fuzi fat-soluble alkaloids
TAAR: Total alkaloids of aconite root
**IC**_**50**_: Half-maximal inhibitory concentration
**EC**_**50**_: Half-maximal effective concentration
b.w.: Body weight
cTnI: Cardiac troponin I
CK-MB: Creatine kinase-MB isoenzyme
CK: Creatine kinase
LDH: Lactate dehydrogenase
cTnT: Cardiac troponin T
AST: Aspartate aminotransferase
ALT: Alanine aminotransferase
ΔΨm: Mitochondrial membrane potential
PGC-1α: Peroxisome proliferator-activated receptor-γ coactivator 1-alpha
NSE: Neuron-specific enolase
AChE: Acetylcholinesterase
SDH: Succinate dehydrogenase
BUN: Blood urea nitrogen
CRE: Creatinine
T: Testosterone
FSH: Follicle-stimulating hormone
LH: Luteinizing hormone
**E**_**2**_: Estradiol
P: Progesterone
ROS: Reactive oxygen species
VT: Ventricular tachycardia
LVSP: Left ventricular systolic pressure
LVDP: Left ventricular diastolic pressure
±dp/dt: Rate of rise/fall of ventricular pressure
AC: Aconitine
MA: Mesaconitine
HA: Hypaconitine
BAC: Benzoylaconine
BMA: Benzoylmesaconine
BHA: Benzoylhypaconine
SOD: Superoxide dismutase
GSH: Glutathione
CAT: Catalase
GPx: Glutathione peroxidase
NOAEL: No observed adverse effect level
ECMO: Extracorporeal membrane oxygenation
BBB: Blood–brain barrier
WEC: Whole-embryo culture
HPLC: High-performance liquid chromatography
UPLC-MS/MS: Ultra-performance liquid chromatography–tandem mass spectrometry
DESI-MSI: Desorption electrospray ionization mass spectrometry imaging
ChP: Chinese Pharmacopoeia
