# Supplementary material for: Toxicology and detoxification processing of Fuzi (Aconitum carmichaelii Debeaux lateral root): a comprehensive review integrating historical perspectives and modern research
Source: Front Pharmacol. 2026 Apr 30;17:1750573. doi: 10.3389/fphar.2026.1750573 (PMC13172728; doi:10.3389/fphar.2026.1750573)
Supplement: Supplementary file 1 [file Table1.doc]

Supplementary Table S1. Original classical descriptions of Fuzi processing methods by dynasty

| Dynasty | Processing methods | Origin |
| --- | --- | --- |
| Han | Removing the peel, breaking into 8 pieces, water processing. | *Jingui Yuhan Fanglüe* (金匮玉函方略) |
| Eastern Han | Selecting large roots, soaking in boy's urine (changing daily, 3 days in summer, 5 days in winter), boiling further in fresh boy's urine until cooked through, removing peel and umbilicus, stringing and drying in shade or sun, storing for use. | *Huatuo Shenfang* (华佗神方) |
| Eastern Jin | Pounding into powder, taking one *Daogui* (ancient measure) dose. | *Zhouhou Beiji Fang* (肘后备急方) |
| Southern & Northern Dynasties | Roasting over fire (willow wood preferred) until cracked, scraping off adventitious roots ('buds'), removing base and tip, splitting finely, burying underground overnight, then drying. OR: Yin processing (阴制): removing tip, peel, and base, slicing thinly, soaking with running water and black beans (*Glycine Semen Nigrum*) for 5 days and nights, straining, then sun-drying. | *Leigong Paozhi Lun* (雷公炮炙论) |
| Northern Song | Carbonizing while retaining some properties (*Shao Cun Xing* processing), grinding into powder. | *Yanglao Fengqin Shu* (养老奉亲书) |
| Northern Song | Raw use: removing peel and tip. Processed use: roasting in ash-fire until cracked, then removing peel and tip. | *Michuan Yanke Longmu Lun* (秘传眼科龙木论) |
| Southern Song | Roasting first until cracked, removing peel and umbilicus, chopping, then baking to dry. | *Shi Bian Liang Fang* (十便良方) |
| Southern Song | Placing in stove flue, soaking hot in boy's urine for 5-7 days, peeling, cutting into 4 pieces, soaking again for 2-3 days, wrapping in damp thick paper, burying in hot ashes for half a day, checking for white 'stars' (crystalline spots) and roasting on a tile until spots disappear. For urgent use: cutting into large pieces, boiling briefly in boy's urine, roasting on a hot tile until cooked. | *Xiao'er Douzhen Fanglun* (小儿痘疹方论) |
| Southern Song | (Similar to above) Placing in stove flue, soaking hot in boy's urine for 5-7 days, peeling, cutting into 4 pieces, soaking again for 2-3 days, wrapping in damp thick paper, burying in hot ashes for half a day, checking for white 'stars' and roasting on a tile until spots disappear. For urgent use: cutting into large pieces, boiling briefly in boy's urine, roasting on a hot tile until cooked. | *Chen Shi Xiao'er Douzhen Fanglun* (陈氏小儿痘疹方论) |
| Southern Song | Roasting (Pao), soaking in salt water, roasting again; repeating this process 7 times; omitting the soaking step on the 7th time, removing peel and umbilicus. | *Sanyin Jiyi Bingzheng Fanglun* (三因极一病证方论) |
| Southern Song | One piece, processed by roasting (Pao), soaking in salt water, roasting again, repeated 7 times; omitting soaking on the 7th time, removing peel and umbilicus. | *Huoren Shizheng Fang Houji* (活人事证方后集) |
| Yuan | Frequently boiling and soaking in boy's urine to neutralize its toxicity. | *Bencao Yanyi Buyi* (本草衍义补遗) |
| Yuan | Wrapping in wet paper, roasting in ashes (Hui Zhong Pao), removing the black peel and umbilicus. | *Zhenzhu Nang Buyi Yaoxing Fu* (珍珠囊补遗药性赋) |
| Yuan | Method for using raw or processed drugs: Fuzi must be roasted (Pao) to control its toxicity. | *Chanbao Baiwen* (产宝百问) |
| Yuan | Washing with medicinal wine and sun-drying. Chuanwu (*Aconiti Radix*) and Fuzi must be roasted (Pao) to control toxicity. | *Yongyao Xinfa* (用药心法) |
| Ming | One piece, roasting (Pao), then soaking in salt water, roasting again, repeating this process 7 times, removing peel and umbilicus. | *Chongke Wan Shi Jiachuan Jishi Liangfang* (重刻万氏家传济世良方) |
| Ming | One Fuzi root, 7 qian, soaking in boy's urine, roasting until cooked, removing peel and umbilicus, grinding into powder, dividing into two doses, decocting each dose with three cups of wine down to one cup before administration. | *Chongke Wan Shi Jiachuan Jishi Liangfang* (重刻万氏家传济世良方) |
| Ming | Processing methods include: boiling in boy's urine; soaking in ginger juice & salt water; using licorice (*Glycyrrhizae Radix et Rhizoma*); using Coptis (*Coptidis Rhizoma*); or combinations. Recommended method: Soak in strong licorice decoction for several days, peel, cut into 4 pieces, soak again for 2-3 days until soft, slice, stir-fry over gentle heat until almost dry but still retaining a pungent taste. For urgent use: wrap in thick paper soaked in licorice decoction and roast (Wei or Zhi), or wrap in dough and roast (Wei). Boiling thoroughly in plain water removes toxicity but also properties; using licorice retains properties while reducing toxicity. Processing should be adequate; improper processing is still toxic. | *Bencao Zheng* (本草正) |
| Ming | One piece, roasted, peel and umbilicus removed, soaking in salt water for a considerable time. | *Bencao Danfang* (本草单方) |
| Ming | Boiling and roasting in boy's urine until cracked and yellow throughout, removing peel and umbilicus before use. | *Shibing Jiefa* (识病捷法) |
| Ming | Processing with boy's urine. | *Bencao Huiyan* (本草汇言) |
| Ming | Processing method: boiling and soaking in boy's urine, then heating over mixed fire (Wen Wu Huo) to reduce toxicity and aid downward action; adding salt enhances the process. | *Chuangyang Jingyan Quanshu* (疮疡经验全书) |
| Ming | Must be roasted until cracked (Pao Che), peel and umbilicus removed, each cut into 8 slices for use. | *Shanghan Xuanlu* (伤寒选录) |
| Ming | Five qian, peel and tip removed, soaking in salt water, wrapping in Mao paper, roasting until half-cooked. | *Jinzai Yiyao* (荩斋医要) |
| Ming | Soaking in boy's urine for 3 days (changing daily), cutting into 4 pieces, soaking again for several days. Roasting one liang. | *Waike Lili* (外科理例) |
| Ming | Processing with boy's urine. | *Bencao Huiyan* (本草汇言) |
| Ming | Selecting large roots (approx. 2 liang each); decocting 4 liang of licorice into a strong soup, soaking the Fuzi for 2-3 days, peeling, cutting into 4 pieces, soaking again for 1 day until thoroughly penetrated, slicing, then stir-frying slowly over low heat until about 70% cooked (avoiding over-frying to preserve properties). | *Leijing Tuyi* (类经图翼) |
| Ming | Eight qian of Fuzi processed with boy's urine, decocting with 4 sheng of water down to 1 sheng, taking the decoction warm. | *Bencao Huiyan* (本草汇言) |
| Qing | Soaking and boiling in boy's urine. | *Xu Ping Waike Zhengzong* (徐评外科正宗) |
| Qing | Large roots (over 1 liang), soaking in boy's urine (changing daily, 3 days in summer, 5 days in winter), boiling until cooked through, removing peel and umbilicus, stringing and drying in shade or sun, storing for use. | *Xu Ping Waike Zhengzong* (徐评外科正宗) |
| Qing | Soaking in half-boiled water ('Sheng Shou Tang') for half a day, wrapping in white ash to dry. OR: Fermenting with rice porridge and yeast/starter. OR: Vinegar processing: prepare vinegar from barley porridge and yeast starter, soak Fuzi roots (after removing rootlets) in the vinegar in a new jar for 7 days (stirring daily), drain and spread on a sieve until white mold appears, then sun-dry slowly for 10+ days until fully dry. OR: Fermentation method: Embedding in fermented vinegar mash (Cu Pei) in a sealed room for a month, then taking out to dry. Mentions Hong Jing's method: Roasting in hot ashes until cracked, avoiding over-charring, except for Jiang Fu Tang (Ginger-Aconite Decoction) where raw Fuzi is used. Stresses the importance of processing with licorice, ginseng, and fresh ginger to control toxicity. | *Binhu Paozhi Fa* (濒湖炮炙法) |
| Qing | Processing Wu Tou (*Aconiti Radix*): Roasting over mixed fire (Wen Wu Huo) until cracked. Processing Fuzi: Select large roots with specific features, roast in willow wood ash fire until cracked, scrape off adventitious roots ('buds'), remove base and tip, break into pieces, bury underground overnight, then dry. Yin processing: remove peel, tip, and base, slice thinly, soak in running water with black beans for 5 days and nights, drain and sun-dry. (Note by Li Shizhen): Raw Fuzi is used for dispersion, processed Fuzi for potent tonification. Raw Fuzi requires Yin processing. Processed Fuzi: Soak in water, roast until cracked, remove peel/umbilicus, slice while hot, then stir-fry until yellow throughout, remove 'fire toxin' (Huo Du). Alternative method: Boil each root with licorice (2 qian), salt water (half cup), ginger juice (half cup), and boy's urine (half cup) until cooked, let sit overnight to remove 'fire toxin'. | *Yaojian* (药鉴) |
| Qing | Yin processing: removing tip, peel, and base, slicing thinly, soaking with running water and black beans (5 liang beans and 6 sheng water per 10 liang Fuzi) for 5 days and nights, then draining and drying. For roasting: Roast in ash fire until skin wrinkles/cracks, scrape off adventitious roots, remove base and tip, split finely, bury underground overnight, then dry. | *Michuan Yinzhi Bencao Dacheng Yaoxing Fu* (秘传音制本草大成药性赋) |
| Qing | Soaking in boy's urine for 7 days, wrapping in dough and roasting (Wei) until cooked. | *Cangsheng Siming* (苍生司命) |
| Qing | Large Fuzi root: soaking and boiling in boy's urine to neutralize toxicity. | *Yimen Mizhi* (医门秘旨) |
| Qing | Selecting large roots (1.3-1.4 qian) with specific features, placing on hot stove or roasting over charcoal, immersing hot in boy's urine for 5-7 days until penetrated, peeling, cutting into 4 pieces, wrapping in thick paper, burying in hot ashes, checking for white 'stars' and roasting on tile until spots disappear. For urgent use: cutting large pieces and roasting on hot tile until cooked. | *Baoying Cuoyao* (保婴撮要) |
| Qing | Large Fuzi root (weighing 1-2 liang), remove peel, wrap in 40-50 layers of wet cotton paper, roast (Wei) over charcoal fire until the paper is dry. | *Yaojian* (药鉴) |
| Qing | Roasting (Pao) in pit ash until slightly cracked, then scraping off the black peel before weighing. Raw use only in Jiang Fu Tang and in plasters or medicinal wines, requires peeling first. Breaking along natural grain into 7-8 pieces, trimming off outer black areas and the tip. | *Bencao Pinhui Jingyao* (本草品汇精要) |
| Qing | Number of roots depends on size (consider half a liang as one piece equivalent), peel removed. Boiling in boy's urine for the time it takes one incense stick to burn (approx. 1 hour). | *Tanhuo Dianxue* (痰火点雪) |
| Qing | Blanching briefly in boiling water, removing peel and umbilicus, cutting into 4 pieces, decocting with 2 zhong (cups) of strong licorice decoction over low heat until the liquid is absorbed, then drying indirectly (e.g., over paper). Or processing with boy's urine is suitable for immediate use but not for storage. | *Bencao Tongxuan* (本草通玄) |
| Qing | Removing peel and umbilicus, first boiling 7 times in a clay pot with half a cup each of salt water and ginger juice. Redness of body and eyes after administration indicates Fuzi toxicity. | *Chishui Xuanzhu* (赤水玄珠) |
| Qing | Prefer roots with black skin, round top, weighing about 1 liang each. Wrapping in dough and roasting in fire (Wei), removing peel and umbilicus, soaking in boy's urine overnight, simmering over low heat, sun-drying, storing in a sealed container, and slicing before use. Raw Fuzi is also used. | *Shoushi Baoyuan* (寿世保元) |
| Qing | Removing peel and umbilicus, first boiling 7 times in a clay pot with half a cup each of salt water and ginger juice, then adding Coptis (*Coptidis Rhizoma*, half liang) and licorice (half liang), adding another half cup of boy's urine, boiling again 7 times, simmering for a while, transferring to a ceramic container, burying underground for a day and night, then retrieving and sun-drying for later use. Prefer round-topped, centrally umbilicated roots weighing 1 liang each. | *Shanghan Liushu Zuanyao Bianyi* (伤寒六书纂要辨疑) |
| Qing | Selecting large roots (1.3-1.4 qian) with specific features (lotus petal shape, round head, flat base). Soaking in boy's urine (5-6 bowls) for 5-7 days until penetrated, peeling, cutting into 4 pieces, soaking again for 3-4 days. Wrapping in several layers of thick paper, moistening, roasting (Wei) in hot ashes, taking out, slicing, checking for white 'stars', and roasting on a new tile until spots disappear. For urgent use: cutting large pieces, boiling 3-4 times in boy's urine, then roasting on a hot tile until cooked. | *Yiguan* (医贯) |
| Qing | General use: Soaking in water, roasting over mixed fire (Wen Wu Huo) until cracked and yellow inside and out, breaking off and discarding the peel and umbilicus before use. | *Bencao Jiyao* (本草集要) |
| Qing | Using boy's urine after boiling it. | *Baochi Quanshu* (保赤全书) |
| Qing | Large Fuzi roots (over 1 liang each, quantity as needed), soaking in boy's urine to cover by 3 cun, changing urine daily (soak 3 days in summer, 5 days in winter), then boiling in fresh boy's urine until cooked through, removing peel and umbilicus, stringing and drying in shade or sun, storing for use. | *Xinkan Waike Zhengzong* (新刊外科正宗) |
| Qing | Fuzi (roasted (Pao), peel removed). One large Fuzi root, roasted and ground into powder, then made into pills using ginger juice as a binder. | *Zhengzhi Yaojue Leifang* (证治要诀类方) |
| Qing | Stir-frying with decocted Fuzi juice. OR: Boiling/soaking in boy's urine, or dough-wrapped roasting, or boiling in Coptis-licorice decoction. | *Shenzhai Yishu* (慎斋遗书) |
| Qing | Removing peel and umbilicus, first boiling 7 times in a clay pot with half a cup each of salt water and ginger juice, then adding Coptis (half liang) and licorice (half liang), adding another half cup of boy's urine, boiling again 7 times, simmering. Retrieving, storing in a ceramic container, burying underground for a day/night, then sun-drying. Select round, centrally umbilicated roots weighing 1 liang each. | *Yixue Xinzhi Quanshu* (医学新知全书) |
| Qing | Soaking Fuzi, then soaking in salt water, repeating the soaking 7 times, removing peel and umbilicus. OR: Preparing a thin paste with boiling water and aged wall clay (Chen Bi Tu), soaking Fuzi in this paste 7 times, dividing the dose. | *Xingyuan Shengchun* (杏苑生春) |
| Qing | Soaking in wine 7 times, removing peel and tip. | *Chen Su'an Fuke Bujie* (陈素庵妇科补解) |
| Qing | Wrapping in dough, roasting (Wei), removing peel and umbilicus, soaking in boy's urine for 3 days, baking dry. Dosage: 3 qian. | *Xuanxiu Bianfang* (悬袖便方) |
| Qing | One piece, boiling in salt water, removing peel and umbilicus. OR: One Fuzi piece, roasting 7 times and soaking in salt decoction 7 times, removing peel and umbilicus, dividing into two doses. | *Yifang Kao Shengqian* (医方考绳愆) |
| Qing | Stir-frying with decocted Fuzi juice. OR: Boiling/soaking in boy's urine, or dough-wrapped roasting, or boiling in Coptis-licorice decoction. | *Zhou Shenzhai Yishu* (周慎斋遗书) |
| Qing | Large Fuzi root: wrapping in wet paper, roasting over fire (Pao) until cracked, removing peel and umbilicus, slicing. | *Renshu Bianlan* (仁术便览) |
| Qing | One piece, soaking in salt water 7 times, removing peel and umbilicus. | *Dantai Yu'an* (丹台玉案) |
| Qing | Fuzi (two roots: one raw, peeled; one soaked in salt decoction, peeled, roasted (Pao)). OR: One Fuzi root, roasted until cracked, soaked in salt water, roasted again, repeat 7 times. If not soaked, remove peel/umbilicus. | *Mingfang Leizheng Yishu Daquan* (名方类证医书大全) |
| Qing | Soaking in salt water, one liang. | *Danxi Zhaixuan* (丹溪摘玄) |
| Qing | Soaking Fuzi in water and salt. | *Cheng Shi Shifang* (程氏释方) |
| Qing | Large Fuzi (5 qian, wrapped in dough and roasted (Wei)). Raw Fuzi (wrapped in dough and roasted (Wei)). One liang each. | *Youke Zhengzhi Zhunsheng* (幼科证治准绳) |
| Qing | Often boiling and soaking in boy's urine to neutralize toxicity and aid downward action; adding salt enhances the process. | *Danxi Xinfa Fuyu* (丹溪心法附余) |
| Qing | One piece, weighing one liang, soaking in boy's urine, baking. | *Dantai Yu'an* (丹台玉案) |
| Qing | One large Fuzi root, peel and umbilicus removed, cutting into large slices, coating with honey and roasting (Zhi) until yellow, then holding in the mouth and swallowing slowly. | *Weisheng Yijian Fang* (卫生易简方) |
| Qing | Roasting (Pao) with boy's urine and yellow mud. | *Zunsheng Bajian* (遵生八笺) |
| Qing | One piece, processed by soaking in salt water and then roasting, repeating this process 7 times; omitting the soaking step on the 7th time, removing peel and umbilicus. | *Michuan Zhengzhi Yaojue* (秘传证治要诀) |
| Qing | One piece, soaking thoroughly in one bowl of ginger juice, then boiling until dry. | *Jianming Yigou* (简明医彀) |
| Qing | One piece, roasting (Wei) over charcoal fire, soaking in salt, roasting again, repeating the soaking 7 times, removing peel and umbilicus, slicing. | *Zhangnüe Zhinan* (瘴疟指南) |
| Qing | Eight fen, stir-frying with Coptis water. | *Yixue Qiongyuan Ji* (医学穷源集) |
| Qing | For ear conditions: Soaking in vinegar and inserting into the ear. Burning to ash. | *Bencao Gangmu (Vol. 1)* (本草纲目上) |
| Qing | For purple and white vitiligo (Zibai Dianfeng): mixing with sulfur (Liu Huang), preparing into a paste with ginger juice, applying by dipping an eggplant calyx (Qie Di) and rubbing onto the affected area. | *Bencao Gangmu (Vol. 1)* (本草纲目上) |
| Qing | One piece, roasting (Pao), soaking in salt water, roasting again, repeating this process 7 times, removing peel and umbilicus. | *Yuji Bianzheng* (玉机辨症) |
| Qing | Eight fen, soaking in salt water until black. | *Shanghan Yuebian -* (伤寒约编 -) |
| Qing | Soaking in boiling water to remove the peel and umbilicus. | *Jingyan Danfang Huibian* (经验丹方汇编) |
| Qing | Taking out (from previous processing), burying in cold ash (Pei). Taking half a liang, grinding together with 1 qian of genuine La Cha (wax tea). Dividing into two doses. For each dose: decocting with 1 zhan (cup) of water down to 6 fen (0.6 zhan), adding half a spoonful of honey just before finishing, letting it cool to warm/cool before taking. Continue until restlessness ceases, followed by sleep and sweating, indicating recovery. | *Zhengzhi Hecan* (证治合参) |
| Qing | Burning to ash while retaining properties (Shao Hui Cun Xing), grinding into powder, taking as a single dose mixed with honey water. | *Leizheng Zhicai* (类证治裁) |
| Qing | One qian, stir-frying with salt water. | *Jianbian Liangfang* (简便良方) |
| Qing | Rinsing lightly to remove pungency, sandwiching between slices of fresh ginger, steaming until thoroughly cooked. | *Mujing Dacheng* (目经大成) |
| Qing | Soaking in boy's urine for one bowl's worth (duration?), steaming, slicing finely, drying. | *Bencao Gangmu Shiyi* (本草纲目拾遗) |
| Qing | Soaking to remove peel and umbilicus. | *Luo Shi Huiyue Yijing* (罗氏会约医镜) |
| Qing | Roasting hot, removing peel, filing into powder. OR: Filing into 8 pieces. | *Shesheng Zonglun* (摄生总论) |
| Qing | Summarizes various methods: Honey-roasting (Mi Zhi); crushing and stir-frying yellow; making pills with ginger juice paste; peeling, breaking into 4 pieces, soaking in red adzuki bean water, hiding Fuzi within, simmering until cooked, removing beans, baking and grinding; soaking in boy's urine for 3 nights (changing daily), rubbing off skin with cloth, pounding to paste, making pills with wine paste; removing peel/umbilicus, slicing; roasting (Pao), removing peel, powdering; mixing Fuzi powder with saliva; roasting (Pao), removing peel/umbilicus, soaking well in salt water; processing (Paozhi), filing into 8 pieces; Raw use: soaking in boy's urine without boiling OR thin slicing after removing peel/umbilicus and soaking in running water + black beans for 5 days/nights, drying. Processed (Shu) use: Best follows Danxi's method (boiling with licorice, salt water, ginger juice, boy's urine), removing fire toxin overnight. | *Bencao Shu Gouyuan* (本草述钩元) |
| Qing | Removing peel and umbilicus, slicing into 4 pieces, mixing with one bowl of strong vinegar, roasting (Zhi) the slices while repeatedly dipping them into the vinegar until all the vinegar is absorbed. | *Jiyin Baofa* (济阴宝筏) |
| Qing | Wrapping Fuzi with 6 liang of crushed old ginger, then encasing in dough/flour cake, further wrapping with 5-7 layers of straw paper, moistening with water, and roasting (Wei) inside a fire for 1-2 Shichen (2-4 hours). Retrieving when cooked, using entirely. Avoiding iron. | *Jiren Baoji* (济人宝笈) |
| Qing | Boiling with fresh ginger. OR: Soaking in boy's urine for 3-4 days until penetrated, wrapping in dough, roasting (Wei) until cooked, removing peel and umbilicus, cutting into 4 pieces with a bamboo knife, boiling together with licorice (Gancao) and Fangfeng (*Saposhnikoviae Radix*) until cooked, then sun-drying. OR: Boiling in boy's urine, removing peel and umbilicus, slicing, baking dry, and grinding finely. | *Qifang Leibian* (奇方类编) |
| Qing | Roasting slowly in ash fire until cracked, removing peel, umbilicus, and tip, then soaking in boy's urine overnight to counteract its "dryness toxicity". OR: Soaking in boy's urine, removing peel, cutting into 4 pieces, then boiling again with boy's urine plus licorice (Gancao) and Fangfeng (*Saposhnikoviae Radix*) until the liquid evaporates. | *Shoushi Qingbian* (寿世青编) |
| Qing | Boiling Fuzi in urine. | *Bencao Chongyuan* (本草崇原) |
| Qing | Processing Fuzi with ginger juice. | *Zhang Shi Yitong* (张氏医通) |
| Qing | Soaking in boy's urine for 3 days, removing peel and umbilicus, cutting into 4 pieces, soaking in licorice decoction for 3 days, wrapping in wet paper and roasting (Wei) until cooked, then burying in ashes for 2 Shifen (approx. 1 hour). Selecting roots weighing at least 1 liang with specific shapes ("squatting posture, square nodes") is preferred. | *Yijing Yunzhong* (医经允中) |
| Qing | Soaking in licorice water for 7 days, changing the water daily. At the end of the period, wrap well in half a jin of dough, roast (Wei) in a charcoal fire until cooked, slice, and bake dry. | *Wanjin Zhibao* (万金至宝) |
| Qing | Half raw and half cooked, processed by roasting (Wei) while wrapped in dough. | *Huizhitang Jingyan Fang* (惠直堂经验方) |
| Qing | Soaking in salt water seven times (or cycles), removing peel, dividing equally, grinding into powder. | *Bencao Shu* (本草述) |
| Qing | Gently roasting (Zhi) with alcohol/ester ('Chun Zhi' 醇酯). | *Buyao Liangfang Xuji* (不药良方续集) |
| Qing | Slicing, soaking in water for two Shichen (4 hours), rinsing briefly. Boiling in honey for 1 day, changing the water and boiling for half a day, adding Ren Shen (*Ginseng Radix et Rhizoma*, 2 liang), decocting to get a concentrated juice, mixing this juice with the Fuzi slices, then sun-drying. (Note: Fuzi must be sliced). | *Yixue Yinlu* (医学引路) |
| Qing | Soaking in honey water for 1 day, boiling for the time it takes 3 incense sticks to burn (approx. 3 hours), baking dry. OR: Processing with boy's urine, then roasting (Zhi) with butter oil (Su You). | *Jiyan Liangfang* (集验良方) |
| Qing | Soaking in boy's urine, removing peel and umbilicus. | *Baihou Bianzheng* (白喉辨证) |
| Qing | Gently roasting (Zhi) Fuzi with wine/vinegar (醇酢), sharpening the tip and inserting it (likely for topical/suppository use). | *Shanju Bencao* (山居本草) |
| Qing | Wrapping in dough, roasting in fire (Wei), removing peel and umbilicus. | *Huoren Xinfa* (Liu Yiren Zhu) (活人心法(刘以仁著)) |
| Qing | Soaking in warm water, washing to remove saltiness, removing outer black peel, slicing. OR: Repeatedly soaking in very hot water until most saltiness is removed, draining, air-drying slightly. Adding 1 jin of Guang Jun Jiang (a type of ginger), roasting over firewood until yellow and cracked open, then slicing. | *Yanke Qishu* (眼科奇书) |
| Qing | First boiling ginger juice vigorously (more than 10 boils), adding Fuzi and boiling further, then removing and letting cool completely. OR: Taking one bowl of vinegar, roasting (Zhi) the Fuzi thoroughly over fire from all four sides until the vinegar has evaporated, removing peel and umbilicus. | *Jingui Qiyao* (Fuke) (金匮启钥(妇科)) |
| Qing | Stir-frying with salt water until black. | *Shanghan Yuebian -* (伤寒约编 -) |
| Qing | Selecting large roots with specific features (lotus petal shape, round head, flat base). Soaking in boy's urine for 5-7 days until penetrated, peeling, cutting into 4 pieces, soaking again for 3-4 days. Wrapping in several layers of thick paper, moistening, roasting (Wei) in hot ashes, taking out, slicing, checking for white 'stars', and roasting on a new tile until spots disappear. For urgent use: cutting into thin large slices, boiling 3-4 times in boy's urine, roasting on hot tile until cooked. Notes on formula compatibility (Ba Wei Wan). | *Qi Shi Yi'an* (齐氏医案) |
| Qing | One large Fuzi root, soaking in warm water for 3 days, drying slightly. Placing on a brick and gradually heating it from all four sides, quenching with fresh ginger juice. Repeating the heating and quenching process until approximately one bowl of ginger juice has been absorbed. Pounding into powder. | *Neike Zhaiyao* (内科摘要) |
| Qing | Raw use: Yin processing (thin slicing, soaking 5 days in running water + black beans, drying). Processed use: soak in water, roast until cracked, remove peel/umbilicus, slice hot, stir-fry until yellow. Alternative: boil with licorice (2 qian), salt water, ginger juice, boy's urine (half cup each) until cooked, remove fire toxin overnight. Market processed Fuzi (Fu Pian): soak 7 days, changing water, scrape peel/umbilicus, boil with licorice, ginger, alum (Bai Fan), drain, slightly dry, store in jars, dry in shade, slice thinly. Newer trade form (Fu Kuai): soak in running water for several days, boil, slice thickly - less potent. | *Bencao Gangmu Yizhi Lu* (本草纲目易知录) |
| Qing | Raw Fuzi powder mixed with scallion juice. OR: Large Fuzi (heavy ones processed with ginger juice, dried in shade, 1 liang), soak thoroughly in Shaojiu (distilled spirit), sun-dry. Soften by soaking in Shaojiu, insert 7 Hongniangzi (Mylabris) into drilled holes, wrap in cotton paper, moisten, roast (Wei) until cooked, remove Hongniangzi, slice, sun-dry powder. Grind chicken liver, sparrow brain, turtle gall together, mix with Fuzi powder, make pills size of Wutongzi. | *Liangpeng Huiji Jingyan Shenfang* (良朋汇集经验神方) |
| Qing | Wrapping in dough, roasting (Wei), removing peel and umbilicus. | *Liangpeng Huiji Jingyan Shenfang* (良朋汇集经验神方) |
| Qing | Prefer round-topped roots weighing 1 liang and several qian. Soaking thoroughly in boy's urine, then boiling with black beans (Hei Dou) for the time it takes 3 incense sticks to burn (approx. 3 hours), then sun-drying. | *Houde Tang Jiyanfang Cuibian* (厚德堂集验方萃编) |
| Qing | Removing peel, slicing to the thickness of a coin, soaking in honey and roasting (Zhi) until yellow; repeating the soaking and roasting process (more repetitions are better), being careful not to char the slices. | *Houke Jiye* (喉科集腋) |
| Qing | Decocting in strong licorice soup to remove toxicity. Processing (Pao) with ginger juice. | *Suixiju Chongding Huoluan Lun* (随息居重订霍乱论) |
| Qing | Removing peel and umbilicus, cutting into large slices, coating with white honey, roasting (Zhi) until thoroughly penetrated and a deep yellow color. | *Waike Zhengzhi Quanshu* (外科证治全书) |
| Qing | Soaking in boy's urine to cover by 3 cun, changing urine daily (soak 3 days in summer, 5 days in winter), then boiling in fresh boy's urine until cooked through, removing peel and umbilicus, stringing and drying in shade or sun, storing for use. | *Waike Dacheng* (外科大成) |
| Qing | Soaking and boiling in boy's urine. OR: Slicing, baking into powder, mixing with vinegar to form cakes. | *Youyou Jicheng* (幼幼集成) |
| Qing | Removing peel/umbilicus, boiling with Chuan Jiao (Zanthoxyli Pericarpium) and licorice (5 qian each) in river water for 3 incense sticks' time, sun-dry. OR: Soaking in boy's urine for 3 days, boiling for 3 incense sticks' time, slice, bake dry. | *Zhongzi Xinfa* (种子心法) |
| Qing | Raw use requires peeling/removing umbilicus. Processed use involves soaking in water, peeling/removing umbilicus completely, slicing while hot, then stir-frying. | *Bencao Yidu* (本草易读) |
| Republic of China (民国) | Pounding into a paste, mixing with scallion juice (Cong Xian). | *Chaoben Yanfang* (抄本验方) |
| Republic of China (民国) | Soaking in boy's urine for 3 days and nights, changing the urine-soaked cloth daily and using it to rub off the peel, pounding into a paste, forming pills the size of small adzuki beans using wine paste as a binder. | *Danfang Jinghua* (丹方精华) |
| Republic of China (民国) | Stir-frying with salt and wine. | *Chongkan Mei Shi Yanfang Xinbian* (重刊梅氏验方新编) |
| Republic of China (民国) | Creating a cavity in the center of the umbilicus (Navel) about the size of a *Gleditsia sinensis* seed, inserting 3 qian of Cinnabar (Zhu Sha), wrapping in wet paper, and roasting (Wei). (Likely external/topical use). | *Nüke Zhengzhi Yuezhi* (女科证治约旨) |
| Republic of China (民国) | Processing Fuzi: Slicing after removing peel and umbilicus, blanching twice consecutively with boiling water to remove saltiness and toxicity, partially drying, then stir-frying in a copper vessel until cooked through for use. Alternatively, soaking in Coptis (Huang Lian) water, then roasting Fuzi over fire (Huo Pao). | *Zhuyuan Yiyao Hekan* (著园医药合刊) |
| Contemporary (当代) | Modern processing overview based on soaking in brine ('Dan Shui Gang' / 'Yan Shui Gang'): Bai Pian (steamed, sun-dried, sulfur-fumigated); Huang Pian (dyed, charcoal dried, sun-dried); Linjiang Pian (refined Huang Pian); Hei Pian (steamed/sunned salt-preserved slices); Gua Pian (split small roots); Pao Pian (planed thin slices); Liu Ye Pian (willow-leaf shape); Hao Pian (roasted, curled); Yan Fuzi (salt-soaked, whole); Gan Fuzi (dried raw); Sheng Fuzi (sliced raw, sulfur-fumigated); Chuan Wu Tou (main root); Fuzi Gao (paste); Fuzi Jing (essence); Fuzi Yan (salt byproduct). | *Yue Meizhong Yi Ji* (岳美中医集) |
| Unknown (不详) | Processing with ginger juice. | *Yi Sheng* (医賸) |
